# Human health implications of trace metal contamination in topsoils and brinjal fruits harvested from a famous brinjal-producing area in Bangladesh

**DOI:** 10.1038/s41598-022-17930-5

**Published:** 2022-08-22

**Authors:** Anika Bushra, H. M. Zakir, Shaila Sharmin, Q. F. Quadir, M. H. Rashid, M. S. Rahman, Supti Mallick

**Affiliations:** 1 Laboratory of Plant Nutrition and Environmental Chemistry, Department of Agricultural Chemistry, Faculty of Agriculture, Bangladesh Agricultural University, Mymensingh, 2202 Bangladesh; 2 College of Agricultural Sciences, International University of Business Agriculture and Technology (IUBAT), Uttara Model Town, Dhaka, 1230 Bangladesh; 3 Department of Agronomy, Faculty of Agriculture, Bangladesh Agricultural University, Mymensingh, 2202 Bangladesh

**Keywords:** Environmental sciences, Natural hazards, Risk factors

## Abstract

A study was undertaken to determine the contents of trace metals in 60 topsoils and 80 brinjal fruits samples from a famous brinjal-producing area of Bangladesh using atomic absorption spectrophotometer. The study also looked at soil pollution levels, dietary intake of nutritionally important trace elements, and human health risks from toxic metals induced by dermal soil exposure and consumption of brinjal. The content of Pb, Ni, Cd, Cu, Fe, Mn, and Zn in brinjal fruits harvested from farmer′s fields ranged from 0.204–0.729, 0.031–0.212, < 0.010–0.061, 1.819–2.668, 3.267–5.910, < 0.010–0.866 and 2.160–3.846 µg g^-1^, respectively, while the amount of Cr was negligible. The calculated enrichment factors showed that 70, 50, and 25% of soil sampling sites had values in the 2.00–5.00 range for Pb, Zn, and Cd, respectively, while 30% of sites had values > 5.00 for Cd, indicating moderate to significant enrichment of these metals in the soil. The study also revealed that brinjal consumption provides a tiny amount of nutritionally important trace elements required for an adult human. Regarding the computed incremental lifetime cancer risks (ILCR), the study revealed that the values for Pb and Ni in all samples and Cd in 40% of samples were several hundred times higher for males and females than the USEPA threshold level due to oral ingestion of brinjal fruits. In contrast, dermal exposures to soil trace elements were within an acceptable range. The PCA results revealed that the contents of Cd, Pb, Ni, and Cu in soils showed strong positive correlations with those elements present in brinjal. The current study suggests future traceability research, focusing on pinpointing potential entry routes for toxic elements into the vegetable food chain.

## Introduction

Trace metals such as lead (Pb), cadmium (Cd), chromium (Cr), copper (Cu), nickel (Ni), zinc (Zn), iron (Fe), manganese (Mn), and others are found in different compartments of the environment. Among these metals, some are considered hazardous (non-essential metals) to human or animal health, even at low concentrations^1^. However, a few of these metals possess some of the metabolic importance of biota (essential metals) and become toxic or poisonous at higher concentrations (Cu, Fe, Zn, Mn, and Ni)^2^. For example, excess intake of Cu is associated with liver damage^3^. Ni is an essential component of urease but possesses risks at higher concentrations. In contrast, even at low concentrations, Pb and Cd are lethal and may induce urologic disorders, bone weakening, high blood cholesterol, and an increased risk of heart disease^4^. Similarly, excess dietary or inhalation exposure to Cr may cause dermatitis, skin ulcerations, allergic asthmatic reactions, bronchial carcinomas, and gastro-enteritis^5^, and Mn may create neurological disorder (manganism), mitochondrial dysfunction and inflammation^6^.

Bangladesh is one of the overpopulated developing countries in Southeast Asia, with a population of around 160 million^7^ and the presence of different trace elements in Bangladeshi foodstuffs is highly concerning. It has been reported that food items in Bangladesh contain a higher amount of various metals and the concentrations are enough to create different health problems for the people^8,9^. The occurrence of trace metals in the field and farm levels through soil contamination and subsequent accumulation in foodstuffs of Bangladesh is very common. Human-induced activities such as prompt industrialization and their waste disposal, wastewater irrigation, sludge application, use of metal contaminated agrochemicals in soils, and inappropriate handling of food during storage and transport were considered major causes of metal contamination in soils and accumulation in foodstuffs in Bangladesh^10–13^.

Brinjal (*Solanum melongena*) is a popular vegetable used in our daily diet (7.28 g/person-day) and is served all around the year^7,14^. According to Naeem and Ugur^15^, dietary intake of brinjal fruits supplies a significant amount of an adult′s daily need for vitamins, minerals, and phenolic compounds. However, many studies at home and abroad suggested that vegetables such as potato, brinjal, arum, amaranth, radish, lady′s finger, and cauliflower are prone to metal accumulation^16,17^.

According to the Bangladesh Bureau of Statistics, brinjal is the second most important vegetable grown in both the summer (9.6% of total vegetable production) and winter (4.7% of total vegetable production) seasons of Bangladesh. Jamalpur district is one of the Bangladesh′s top producers of brinjal fruits, accounting for around 8.5 percent of the country′s total production, and Islampur Upazila produced the highest amount (60.4 percent of the total output) of brinjal fruits^14^.

The nutritional quality and value of brinjal are well-studied. However, a corroborative study to understand the status of trace metals in brinjal-producing soils and edible parts of brinjal has not yet been conducted for Jamalpur districts in Bangladesh. Furthermore, most of the previous studies related to different metal contamination in the soils of Bangladesh emphasized measuring the level of pollution and ignored assessing potential human health risks due to dermal exposure to those metals. Therefore, this study aims (i) to determine concentrations of different trace elements (Pb, Ni, Cd, Cu, Cr, Fe, Mn, and Zn) in topsoils and brinjal fruits harvested from two densely cultivated Upazila′s of Jamalpur district, Bangladesh, (ii) to evaluate soil pollution level, (iii) to compare the measured dietary intakes of nutritionally important trace metals with recommended dietary allowances (RDA), and (iv) to assess the carcinogenic and non-carcinogenic human health risk associated with oral consumption of brinjal fruits and dermal contact of brinjal-producing soils of Jamalpur district, Bangladesh.

## Materials and methodology

### Soil and brinjal sampling

**Figure 1:**
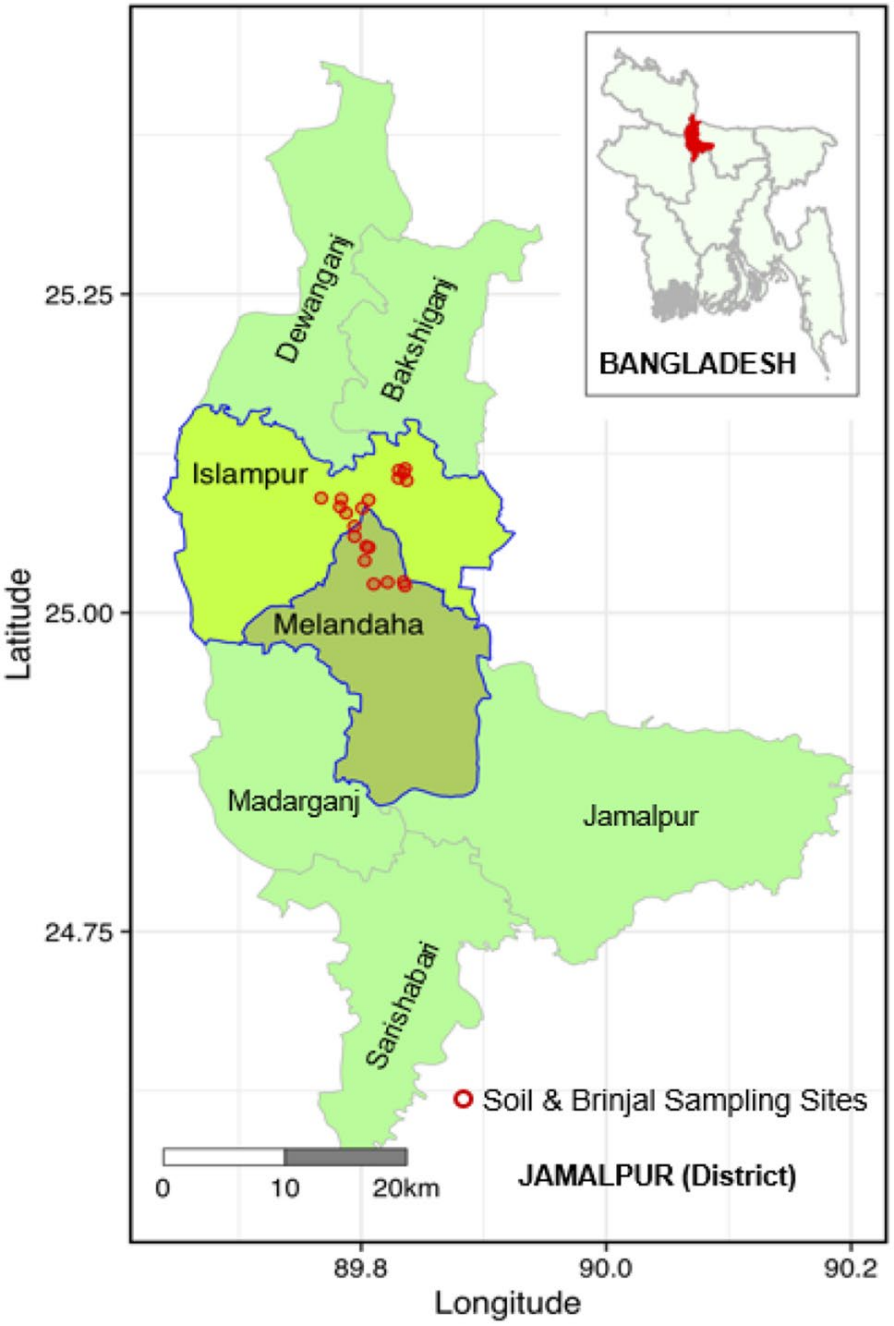
Map shows the soil and brinjal sampling locations of the farmers′ fields of Melandaha and Islampur Upazila of Jamalpur district, Bangladesh.

As illustrated in Fig. 1, two well-known brinjal-producing Upazilas, namely- Islampur and Melandaha of Jamalpur district, Bangladesh, were chosen, with 11 and 9 locations, respectively, representing intensively cultivated areas. Three (3) topsoils (depth, 15 cm) and four (4) brinjal fruits were taken directly from the same location of the fields. Thus, this study handled a total of 60 (20 × 3) soil and 80 (20 × 4) brinjal fruit samples. Sampling was accomplished following the methods mentioned by Tandon^18^.

### Sample processing

In December 2020, soil and brinjal samples were collected directly from the same location in farmers' fields. The samples were then placed in an airtight zipper bag with unique codes and placed in an icebox to maintain a low temperature. Finally, all the collected samples were brought to the Laboratory of Plant Nutrition and Environmental Chemistry, Department of Agricultural Chemistry, Bangladesh Agricultural University (BAU), for further processing and chemical analyses. After homogenization, both brinjal and soil samples were air-dried first and then oven-dried at 50 °C till they reached a constant weight. To avoid cross-contamination, oven-dried samples were thoroughly ground using a clean grinding tool and stored in zipper bags with proper labels and specific code numbers until subsequent chemical analysis. The plant experiments (analysis of brinjal fruits) were performed in accordance with relevant guidelines and regulations.

### Preparation of extracts

Soil samples were extracted in 15 mL Teflon (PTFE) containers for total trace metal contents following the protocol of Tessier et al.^19^ with minor adjustments as mentioned by Zakir and Shikazono^20^. In the case of brinjal fruit, an acid mixture (HNO_3_ and HClO_4_ at a ratio of 2:1) was used to complete the digestion in a block digester (DK 20, VELP Scientifica, Italy)^18^.

### Determination of trace metals and soil physicochemical properties

Eight (8) trace metals, namely-Pb, Ni, Cd, Cu, Cr, Fe, Mn, and Zn, were determined in both extracts by an atomic absorption spectrophotometer (AAS) equipped with a highly sensitive background correction system (SHIMADZU, AA-7000, Japan) at the Department of Agricultural Chemistry, Bangladesh Agricultural University, Mymensingh-2202, Bangladesh. Thousand (1000) µg mL^-1^ stock solution, which was provided by Sigma-Aldrich, USA, was used to prepare standard series solutions for all trace metals. The instrument′s lowest detection limit for all trace metals was 0.01 µg g^-1^. Details of calibration of AAS during operations are presented in Table 1 (Suppl.). However, the determinations of soil physicochemical properties viz. pH, EC and organic carbon (OC) were accomplished following the methods mentioned by Tandon^18^.

### Quality control in the experiments

Two (2) certified reference materials (CRM), namely JSd-1 (Stream sediment) and 7502-a (White rice powder), were used in the present study, and the same procedure was employed to determine the amounts of different trace metals in the extracts of the CRMs to assess the effectiveness of the analytical processes. Table 1 displays the obtained values as well as their percent recoveries. In order to minimize errors in digestion, a blank was also prepared in each case. Additionally, all operations were completed with analytical reagent (AR) grade quality acids (Sigma-Aldrich, USA).

**Table 1:** Analytical results obtained for Certified Reference Material (CRM) samples along with per cent recovery.

Trace metals	Reference # 7502-a (White rice powder)			JSd-1 (Stream sediment)		
	Certified value (µg g^-1^)	Observed value (µg g^-1^)	Recovery (%)	Certified value (µg g^-1^)	Observed value (µg g^-1^)	Recovery (%)
Cu	3.02	3.08 ± 0.060	102.0	22.00	22.62 ± 1.21	102.8
Zn	26.00	24.62 ± 0.041	94.7	96.50	104.22 ± 0.48	108.0
Ni	0.39	0.414 ± 0.008	106.0	7.04	7.66 ± 0.10	108.8
Fe	4.48	4.93 ± 0.071	110.0	–	20,010 ± 511	–
Mn	11.20	10.18 ± 0.085	90.9	–	488.9 ± 2.24	–
Pb	0.0043	bdl (< 0.01)	–	12.90	13.80 ± 4.38	107.0
Cr	0.075	0.084 ± 0.036	112.0	21.50	20.36 ± 1.22	94.7
Cd	0.548	0.522 ± 0.18	95.3	0.146	0.134 ± 0.08	91.8

*bdl*  below detectable limit.

### Assessment of soil pollution level

Enrichment factor (EFc) is an extensively used metric for determining the degree of change in soil characteristics, which is derived as follows:

1 $${EF}_{c}=\frac{({C}_{M}/{C}_{Fe}{)}_{Sample}}{({C}_{M}/{C}_{Fe}{)}_{Eart{h}^{^{\prime}}s\, crust}}$$

where, (C_M_/C_Fe_)_sample_ = The ratio of metal concentration to Fe content in a soil sample and (C_M_/C_Fe_)_Earth’s crust_ = The same reference ratio in the Earth′s crust. The crustal average value of different metals was derived from Taylor^21^. Iron was selected as the benchmark metal because of its prevalence in the upper crust and strong immobility. After the measurement, enrichment levels in soils were categorized following the class mentioned by Barbieri^22^.

### Calculation of human health risk

#### Calculation of daily intake of trace metals through brinjal consumption

The daily intake of trace elements through the dietary consumption of brinjal was estimated using the following equation-

2 $$\begin{gathered} Daily \;intake \;of\; metals\; \left( {\mu g\ day^{-1} } \right) = [Daily\; brinjal\; consumption \left( g \right) \times \hfill \\ \qquad \qquad Metal \;concentration \;in\; brinjal \left( {\upmu \text{g} \,g^{ - 1} } \right)] \hfill \\ \end{gathered}$$

#### Calculation of chronic daily intake (CDI) of trace metals

The CDIs (mg kg^-1^ day^-1^) of trace metals for dietary consumption of brinjal fruits and dermal adsorption of those metals of the brinjal cultivating soils were computed using the USEPA's exposure model^23^ to measure cancer and non-cancer risks.

3 $${CDI}_{Oral}\left(\mathrm{mg }\,{kg}^{-1}{day}^{-1}\right)=\frac{\left(BIR\times {C}_{brinjal}\times EF\times ED\right)}{BW\times AT}$$

4 $${CDI}_{Dermal}\left(\mathrm{mg }\,{kg}^{-1}{day}^{-1}\right)=\frac{\left({C}_{soil}\times CF\times AF\times {ABS}_{d}\times EF\times ED\times EV\times SA\right)}{BW\times AT}$$

The details of variable inputs used in the aforementioned calculations are summarized in Table 2. However, in the calculation of brinjal intake rate (BIR), this study considered total postharvest damage of brinjal 29.4%, which is subtracted from the total production of 557,787 metric tons^24^ and population under 6 years was estimated 10% of total population^25^.

**Table 2:** Input assumptions used to calculate non-carcinogenic human health risk due to different trace metal exposures through dietary intake of brinjal and dermal adsorption of soils.

Parameter	Unit	Values		Reference
		Ingestion	Dermal adsorption	
Metal concentration (C_brinjal_ and C_soil_)	µg g^-1^	–	–	–
Brinjal ingestion rate (BIR)	g person^-1^ day^-1^	7.28	–	^24,25^
Skin surface area available for contact (SA)	cm^2^	–	5700	^23^
Dermal absorption fraction (ABS_d_) for metals	–	–	0.001	^23,26^
Adherence factor of soil to skin (AF)	mg cm^-2^-event	–	0.07	^23^
Exposure frequency (EF)	Days year^-1^	365	350	^23^
Exposure duration (ED)*	Year	Male = 64.8; Female = 67.8	30	^23,24^
Conversion factor (CF)	kg mg^-1^	–	10^–6^	^23^
Average body weight (BW)	kg	Male = 70; Female = 50	Male = 70; Female = 50	^24^
Event frequency (EV)	(events day^-1^)	–	1	^23^
Averaging time (AT)	Days	Male = 23,652; Female = 24,747	10,950 & 25,550 days for non-carcinogenic and carcinogenic effects, respectively	^23,24^

*ED is calculated by deducing the childhood period of 6 years from the total life expectancy.

#### Calculation of non-cancer health risk

The non-cancer human health risks of different trace metals were measured using the following model of USEPA^23^

5 $${HQ}_{Oral}=\frac{{CDI}_{Oral}}{{R}_{f}{D}_{Oral}}$$

6 $${HQ}_{Dermal}=\frac{{CDI}_{Dermal}}{{R}_{f}{D}_{Dermal}}$$

where, HQ refers hazard quotients, and R_f_D indicates reference dose. However, the R_f_D_Oral_ values for different metals were taken from the literature, while R_f_D_Dermal_ values were measured following USEPA′s derivation methodology^23^. Both the R_f_D_Oral_ and R_f_D_Dermal_ values for different trace metals are presented in Table 3.

7 $${{R}_{f}D}_{Dermal}={R}_{f}{D}_{Oral}\times {ABS}_{GI}$$

where, *ABS*_*GI*_ means the fraction of contaminant/ toxicant absorbed in the gastrointestinal tract, and the values for different trace metals were taken from USEPA^23^ and other literature as mentioned in Table 3.

**Table 3:** Oral and dermal reference doses (R_f_D_Oral_ and R_f_D_Dermal_) for different trace elements along with cancer slope factor (CSF).

Metals	Reference dose (mg kg^-1^ day^-1^)		% Absorbed ABS_GI_	CSF_Oral_ (mg kg^-1^ day^-1^)	CSF_Dermal_ (mg kg^-1^ day^-1^)
	R_f_D_Oral_	R_f_D_Dermal_			
Pb	0.0036^27^	0.00108	30.0^28^	0.0085^29^	0.0283
Ni	0.02^30^	0.0008	4.0^23^	0.91^29^	22.75
Cd	0.001^30^	0.000025	2.5^23^	15.0^29^	600
Cu	0.04^30^	0.012	30.0^31^	–	–
Cr	0.001^27^	0.000013	1.3^23^	–	–
Fe	0.70^30^	0.42	60.0^32^	–	–
Mn	0.14^30^	0.0056	4.0^23^	–	–
Zn	0.30^30^	0.06	20.0^33^	–	–

#### Calculation of carcinogenic health risk

The incremental lifetime cancer risk (ILCR) was calculated to determine the risk of carcinogenic health effects from trace metal exposure by soil dermal adsorption and oral consumption of brinjal fruits. The following equations, as defined by the USEPA^23^, were used to compute ILCR values for various trace elements.

8 $${ILCR}_{Oral}={CDI}_{Oral}\times {CSF}_{Oral}$$

9 $${ILCR}_{Dermal}={CDI}_{Dermal}\times {CSF}_{Dermal}$$

The oral cancer slope factor (CSF_Oral_) values for Pb, Ni, and Cd were considered 0.0085, 0.91, and 15.0 mg kg^-1^ day^-1^, respectively^29^. On the other hand, CSF_Dermal_ values for these metals were calculated following USEPA′s derivation methodology^23^, and the obtained results are presented in Table 3. The total ILCR was calculated considering both the oral and dermal CDIs of these trace elements, and the tolerable range was considered 1.0 × 10^–6^ to 1.0 × 10^–4^ for a single carcinogenic agent^34^.

10 $${CSF}_{Dermal}=\frac{{CSF}_{Oral}}{{ABS}_{GI}}$$

### Statistical analysis

The data analyses were carried out using the statistical package ‘R’^35^. The data were tested for normality using the Shapiro–Wilk method before the statistical analyses. Non-parametric Kruskal–Wallis tests were performed for mean comparison. Spearman’s rank-order correlation method was used to evaluate the correlations between metal concentrations in soils and brinjal fruits grown on the respective soils. The relationship pattern of the data set was examined in this study via principal component analysis (PCA) in statistical software Minitab 17 (Minitab Inc., State College, Pennsylvania, USA).

### Ethical approval

All studies were conducted in accordance with relevant guidelines and regulations for the brinjal samples, which were collected directly from the farmers′ field of the study area. This article does not contain any studies involving human and animal participants performed by any of the authors. The manuscript in part or in full has not been submitted or published anywhere.

## Results and discussion

### Physicochemical properties of soils

Among the physicochemical properties, pH, electrical conductivity (EC), and organic carbon (OC) contents in topsoils (0–15 cm) of the study regions were measured. The calculated pH, EC, and OC ranged from 5.94 to 6.96, 72.6 to 276.0 µS cm^−1^, and 0.13 to 1.16%, respectively (Table 4). The study revealed a slightly acidic nature of soils, which might be due to plant residue or organic matter decomposition and then organic acid formation^36^. Among the various factors, soil pH is considered an important one, and the acidic nature of soil greatly influences the availability of heavy metals^37^. Similarly, the solubility of different metallic compounds depends on the fraction type of metals, particularly the form of oxides, hydroxides, carbonates, or mineral bound fractions are highly mobile in the acidic pH of the soil^38^. Soil EC is a suggestive result about soil salinity, and according to obtained results, the soils of the study area can be classified as non-saline (EC ≤ 2000 µS cm^-1^), i.e., the salinity effect in all sampling sites was negligible^39^. However, soil OC is another important index that controls the content of metals, the bio-availability, and the chemical behaviour of trace elements. Li et al.^40^ reported that soil OC showed a significant positive correlation with different metals. A higher amount of OC in the soil signifies that trace elements are firmly bound to OC and form metal chelate complexes, resulting in less metal availability for plants^41^. Thus, it can be inferred that a slightly acidic nature and comparatively lower amount of OC in the soil of the study area have a potential influence on the bio-availability of different trace elements.

**Table 4:** Trace metal contents (in µg g^-1^) in soils and brinjal fruits collected from farmers′ fields of Jamalpur district, Bangladesh along with physicochemical properties of soils, and daily intake and recommended dietary allowances of nutritionally important elements.

Parameters	Soil				Brinjal fruit				Daily intake of metal (µg day^-1^)	RDA^a^ (mg day^-1^ person^-1^)	UTIL^a^ (mg day^-1^ person^-1^)
	Min	Max	Mean ^b^	Median	Min	Max	Mean ^b^	Median			
pH	5.94	6.96	6.41	6.30	–	–	–	–	–	–	–
EC (µS cm^-1^)	72.6	276.0	131.0	112.1	–	–	–	–	–	–	–
OM (%)	0.23	2.01	1.27	1.32	–	–	–	–	–	–	–
Pb	9.10	23.66	16.93 (60)	16.32	0.204	0.729	0.431 (80)	0.393	3.14	ND	0.22^42^
Ni	14.05	25.08	20.84 (60)	21.25	0.031	0.212	0.115 (80)	0.108	0.84	ND	1.00^43^
Cd	< 0.01	0.67	0.32 (33)	0.53	< 0.010	0.061	0.018 (32)	0.043	0.13	ND	0.06^42^
Cr	45.76	75.28	62.63 (60)	63.77	< 0.010	< 0.010	< 0.010 (0)	< 0.010	–	M = 0.035, F = 0.025^43^	0.06^42^
Cu	21.67	48.79	31.81 (60)	28.42	1.819	2.668	2.189 (80)	2.130	15.94	M = 0.90, F = 0.90^43^	10.00^43^
Fe	27,126	36,304	31,882 (60)	31,169	3.267	5.910	4.673 (80)	4.833	34.02	M = 8.0, F = 18.0^43^	45.00^43^
Mn	406.6	604.0	471.6 (60)	460.6	< 0.010	0.866	0.231 (68)	0.172	1.68	M = 2.3, F = 1.8^43^	11.00^43^
Zn	67.49	105.40	79.44 (60)	80.82	2.160	3.846	2.685 (80)	2.550	19.55	M = 11.0, F = 8.0^43^	40.00^43^

^a^Life stage group 19–50 years; *RDA*  recommended dietary allowances, *UTIL*  upper tolerable daily intake level, *ND*  not determined, *M*  males, *F*  females. ^b^Value in parenthesis indicates the number of the samples that were above the limit of detection (LoD).

### Trace metal contents in the surface soils

**Figure 2:**
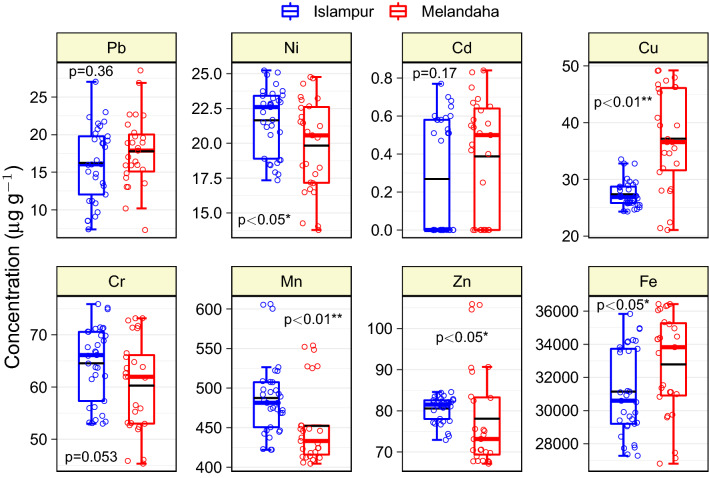
Trace elements concentration in different farmers′ fields soils of brinjal producing areas of Islampur and Melandaha Upazila of Jamalpur district, Bangladesh. Lower and upper box boundaries are 25th and 75th percentiles, respectively, whereas the colored (blue or red) horizontal line inside the box is median and the black line represents the mean value. The lower and upper error lines are 10th and 90th percentiles, respectively. Data points falling outside 10th and 90th percentiles are the outliers. The p-values are mean comparison (nonparametric Kruskal–Wallis test) of soil metal concentrations sampled from two study locations. Single and double asterisks associated with the p-values designate the means are different at 5% and 1% level of probability, respectively.

The present study assessed the contents of several trace elements in topsoils (0–15 cm) of the farmers′ fields of the study regions of Jamalpur district, and to our acquaintance, this is the pioneer report on trace metal content in topsoils of the study area. However, our previous study reported on the content of As in the mentioned area^9^. The present study revealed that the concentrations of Pb, Ni, Cd, Cr, Cu, Fe, Mn, and Zn in soil varied widely among the sampling locations ranging from 9.10–23.66, 14.05–25.08, < 0.01–0.67, 45.76–75.28, 21.67–48.79, 27,126–36,304, 406.6–604.0 and 67.49–105.40 µg g^-1^, respectively (Fig. 2 and Table 4). The mean concentration of trace elements in soils of the study area were in the sequence of Fe > Mn > Zn > Cr > Cu > Ni > Pb > Cd. Among the trace metals studied, Ni, Cu, Mn, Zn and Fe contents in soils differ significantly between the two locations (Fig. 2). The study results revealed little bit higher amounts of Pb (17.80 µg g^-1^), Cd (0.39 µg g^-1^), Cu (37.20 µg g^-1^) and Fe (32,789 µg g^-1^) in soils of Melandaha Upazila compared to Islampur Upazila (16.20, 0.27, 27.40 and 31,141 µg g^-1^, respectively). On the other hand, the amounts of Ni, Cr, Zn, and Mn were comparatively higher in soils of Islampur Upazila (Fig. 2 and Table 3 Suppl.), and such types of little deviations in trace elements content were mainly due to lithological variations in the formation of the soil. Taghipour et al.^44^ also stated that trace metal content could be quite variable in locations with heterogeneous lithology, with the diversity being just a consequence of the parent material and soil characteristics. However, according to Moslehuddin et al.^45^, the contents of Pb, Ni, Cr, Cu, Fe, Mn, and Zn in topsoils of 10 soil series of Bangladesh varied between 30.0–42.0, 8.0–92.0, 24.0–86.0, 8.5–43.3, 9200–47,600, 122–590 and 18.9–92.3 µg g^-1^, respectively, and in most cases present study results were also within these ranges. Rahman et al.^46^ reported that Pb, Ni, Cu, and Fe concentrations in agricultural soils of the Jessore district varied from 0.26–5.44, 2.41–58.35, 1.71–118.05, and 5900–46,000 µg g^-1^, respectively. Kormoker et al.^47^ collected agricultural field soils from 58 sites of the Jhenaidah and Kushtia districts, Bangladesh, and the mean concentration of Pb, Ni, Cd, Cr, and Cu was 19.20, 21.00, 1.20, 5.78, and 31.80 µg g^-1^, respectively. On the other hand, Chowdhury et al.^48^ analyzed 1209 paddy soils collected from 57 Upazilas (sub-districts) of 17 districts in Bangladesh and found that the mean concentrations of Pb, Ni, Cu, Fe, and Zn were 18.0, 41.0, 32.0, 28250, and 70.0 µg g^-1^, respectively. For comparison with geochemical background concentration, the mean concentrations of all trace elements studied were lower than the average shale value^49^, soil toxicity reference values^50^, and soil quality guideline values of Canada^51^, and the Netherlands^52^ (Table 2 Suppl.). However, the average contents of Pb and Cd in soils of the study regions were higher than the crustal average values^21^ and the upper continental crust benchmark values mentioned by Yaroshevsky^53^ (Table 2 Suppl.). In China, Shi et al.^54^ classified the agricultural soils into five areas and reported an elevated concentration of Pb than the national soil background value. They also concluded that Pb was incorporated into agricultural soils from outside sources linked to human activities. The higher levels of Pb and Cd in the study area's soils could be attributed to fluctuations in trace metal concentrations in irrigation water and other agronomic operations in the area. Furthermore, the agricultural soil in Bangladesh is contaminated with trace metals due to recurrent irrigation with wastewater and other sources, as well as the use of inorganic fertilizers and synthetic pesticides^13,55^. Pb and Cd, for example, can be found in irrigation water^56^, and Cd is present in phosphatic fertilizers because it is a contaminant in all phosphate rocks^57^.

The uptake of trace elements from soil to plant, on the other hand, is influenced not only by overall metal concentrations but also by other factors^58^. As a result, a high total trace metal concentration in one location may not be hazardous when contrasted to a low metal concentration in another. The advanced methods for total trace element risk and hazard assessments in surface soil are still in their early stages of development. Thus, future studies should focus on synchronizing soil physicochemical parameters with plant genomics to identify the disadvantages of worldwide comparisons on trace element pollution in the topsoil of farmers' fields.

### Trace metal contents in edible part of brinjal

**Figure 3:**
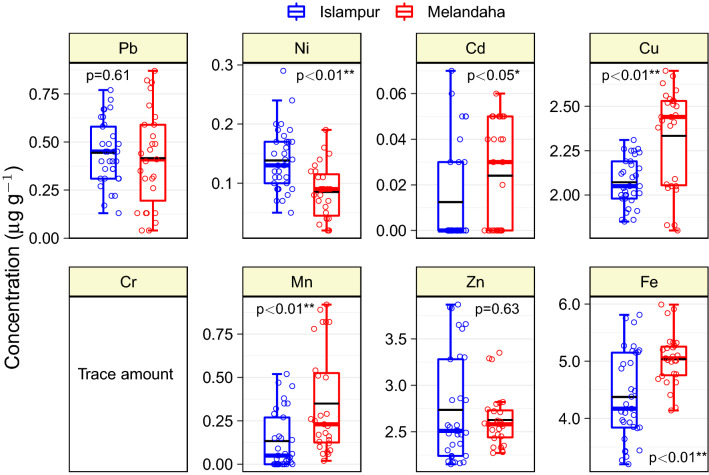
Trace elements concentration in brinjal fruits collected from different farmers′ fields of Islampur and Melandaha Upazila of Jamalpur district, Bangladesh. Lower and upper box boundaries are 25th and 75th percentiles, respectively, whereas the colored (blue or red) horizontal line inside the box is median and the black line represents the mean value. The lower and upper error lines are 10th and 90th percentiles, respectively. Data points falling outside 10th and 90th percentiles are the outliers. The p-values are mean comparison (nonparametric Kruskal–Wallis test) of brinjal metal concentrations sampled from two study locations. Single and double asterisks associated with the p-values designate the means are different at 5% and 1% level of probability, respectively.

The content of Pb, Ni, Cd, Cu, Fe, Mn, and Zn in brinjal fruits harvested from the study regions of Jamalpur district ranged from 0.204–0.729, 0.031–0.212, < 0.010–0.061, 1.819–2.668, 3.267–5.910, < 0.010–0.866 and 2.160–3.846 µg g^-1^ with the mean value of 0.431, 0.115, 0.018, 2.189, 4.673, 0.231 and 2.685 µg g^-1^, respectively (Fig. 3 and Table 4). The study results revealed that all brinjal fruits harvested from the study regions possessed a tiny amount (< 0.010 µg g^-1^) of Cr. Similarly, 60% (44.4% and 88.8% of samples from Melandaha and Islampur Upazila, respectively) and 15% (all from Islampur Upazila) samples also contained negligible amounts (< 0.010 µg g^-1^) of Cd and Mn, respectively (Table 4 Suppl.). With respect to Ni, Cd, Cu, Mn, and Fe contents, there were significant differences observed between brinjal fruit samples collected from Islampur and Melandaha upazila of Jamalpur district (Fig. 3). The average concentration of trace elements in brinjal fruits were in the order of Fe > Zn > Cu > Pb > Mn > Ni > Cd > Cr. So far, we know, there is no study report yet, which is collected brinjal fruits directly from the producers/farmers of well-known brinjal cultivating areas of Bangladesh. Most of the previous studies gathered brinjal fruits from various marketplaces (at the retailer level)^59,60^, and or samples that were grown in contaminated sites^17,55,61^, thus in most cases elevated concentrations of Pb, Cd, Cu, Ni, Cr and Zn were reported when compared to this study. However, a few samples had greater levels of toxic metals (Pb, Cd, and Ni), which could be due to the abuse of toxic metal-containing insecticides during the brinjal's fruiting period. Gimeno-Garcia et al.^62^ reported that inorganic fertilizers and pesticides contained a substantial amount of different trace elements, including Pb, Cd, and Ni. Brinjal producers in our country used a variety of pesticides almost daily from the early fruit setting stage to harvesting, perhaps supplementing trace metals in the fruits^63^.

Table 4 shows the daily intake of trace elements due to consuming brinjal fruits as a vegetable, RDA values, and upper tolerable daily intake levels (UTIL) of metals. The National Academy Press determined the RDA values for nutritionally important trace elements (Cu, Fe, Mn, and Zn)^43^. However, the present study revealed only 1.8% Cu (for both males and females), 0.43% and 0.19% Fe, 0.07% and 0.09% Mn, and 0.18% and 0.24% Zn of total RDA as prescribed for males and females, respectively provided from brinjal, which seems insufficient. This finding suggests that the country's population may be deficient in these nutritionally important trace elements. Hence, a whole diet evaluation and human biomonitoring study will be required in the future to thoroughly assess whether people in the country are at risk of insufficiency or overexposure to nutritionally important trace elements.

On the other hand, regarding toxic metals (Pb, Cd, and Ni), the calculated daily intakes were 3.14, 0.13, and 0.84 µg day^-1^, respectively (Table 4). According to the Joint FAO/WHO Food Standards Programme, permissible limits of Pb and Cd in vegetable samples are 0.30 and 0.05 µg g^-1^, respectively^64^. Considering these values, the present study revealed that 75% and 10% of brinjal fruits samples exceeded the prescribed limit of Pb and Cd, respectively (Table 4 Suppl.), hence may be problematic for human health. However, when we compared with UTIL of Pb and Cd recommended by the AMEC^42^, the contents in brinjal fruits were within the limit (Table 4). On the other hand, the Ni contents in the brinjal fruit samples were within the permissible limit prescribed by the Joint FAO/WHO Food Standards Programme (10 µg g^-1^) and UTIL. This finding suggests that the country's population may be safe as regards Ni content in brinjal. However, a more thorough and critical quantitative investigation should be conducted, taking into account all stakeholders in the distribution network as well as the total diet, to determine the actual status of trace element deficiency or excessive exposure, which will eventually lead to better agricultural practices and food safety in Bangladesh.

### Evaluation of soil pollution

The assessment of trace element pollution status in topsoils of the study regions was done based on enrichment factor (EFc) values. The measured EFc values for Pb, Ni, Cd, Cr, Cu, Mn, and Zn in topsoils of the study regions ranged from 1.33–3.79, 0.39–0.67, 0.08–6.81, 0.94–1.36, 0.61–1.73, 0.66–1.15, and 1.55–2.44, respectively (Table 5). Typically, EFc values less than 1.00 means natural/ normal metal enhancement, but the values more than 1.00 suggest enhancement from the various influence of human activities^65^. Alternatively, Zhang and Liu^66^ reported that EFc = 0.50–1.50 suggests a considerable amount of the trace metal in the soil came through geogenic weathering processes, and EFc value of more than 1.50 indicates a substantial metal content came from the various influence of human activities. Hence, considering the later class, 100%, 80%, 55%, and 5% of soils of the study area had EFc values more than 1.50 for Zn, Pb, Cd, and Cu, respectively, which indicate anthropogenic sources of these trace elements to the soil. Furthermore, 70%, 50%, and 25% of topsoil of the study region possessed EFc values 2.00–5.00 for Pb, Zn, and Cd, respectively, indicating moderate enrichment of these metals in the soil. Additionally, 30% of the locations had EFc values > 5.00 for Cd, indicating significant enrichment of this toxic metal in the study region′s topsoils. However, different anthropogenic activities such as inorganic fertilizers (i.e., phosphatic fertilizer) and pesticides used in farm areas may enrich Cd, Pb, and other trace elements in the soil^57,62^. Hence, thorough studies addressing all agro-ecological zones of the country should be conducted to determine the amounts of toxic compounds, especially metals, in order to help us preserve soil quality and safe agricultural production.

**Table 5:** Summary statistics for enrichment factors (EFc) calculated for selected trace elements in soils of brinjal-producing areas of Jamalpur district, Bangladesh.

Trace metals	Melandaha Upazila		Islampur Upazila	
	EFc values	Type of enrichment	EFc values	Type of enrichment
Pb	1.33–3.59 (2.50)	Anthropogenic	1.35–3.79 (2.37)	Anthropogenic
Ni	0.39–0.51 (0.45)	Natural/ normal	0.45–0.67 (0.52)	Natural/normal
Cd	0.00–5.89 (3.20)	Anthropogenic	0.00–6.81 (2.63)	Anthropogenic
Cr	0.95–1.15 (1.03)	Natural/ normal	1.04–1.36 (1.17)	Natural/normal
Cu	0.61–1.73 (1.18)	Natural/ normal	0.74–1.21 (0.91)	Natural/normal
Mn	0.66–1.05 (0.82)	Natural/ normal	0.74–1.15 (0.93)	Natural/normal
Zn	1.55–2.34 (1.92)	Anthropogenic	1.89–2.44 (2.09)	Anthropogenic

Value in parenthesis indicates the study average.

### Evaluation of human health risk

#### Non-cancer health risk

The total trace metal content in diverse types of Bangladeshi soils has been thoroughly examined^10,11,45–47,61^, but soils trace metal adsorption through dermal route was mostly ignored. Hence, one of the major goals of our research was to assess both the non-cancer and cancer risk due to dermal exposure to different trace metals in the soils of Jamalpur, Bangladesh. The hazard quotient (HQ) values were used to calculate non-carcinogenic human (both adult males and females) health hazards from dermal contact to topsoils in the study regions and consumption of brinjal fruits. A good number of published research studies worldwide used HQ analysis as a key instrument for determining the non-cancer risks caused by the ingestion of hazardous metal-rich foods^8,26,56,59,61^. The average calculated CDI_Dermal_ values for Pb, Ni, Cd, Cu, Cr, Fe, Mn and Zn were 9.25E-08, 1.14E-08, 1.76E-09, 1.74E-07, 3.42E-07, 1.74E-04, 2.58E-06 and 4.34E-07 mg kg^-1^ day^-1^ for adult males, and 1.30E-07, 1.59E-08, 2.47E-09, 2.43E-07, 4.79E-07, 2.44E-04, 3.61E-06 and 6.08E-07 mg kg^-1^ day^-1^ for females, respectively (Table 6). On the other hand, the mean measured HQ_Dermal_ values for Pb, Ni, Cd, Cu, Cr, Fe, Mn, and Zn were 8.57E-05, 1.42E-04, 7.05E-05, 1.45E-05, 2.63E-02, 4.15E-04, 4.60E-04, and 7.24E-06 for males, and 1.20E-04, 1.99E-04, 9.87E-05, 2.03E-05, 3.69E-02, 5.81E-04, 6.44E-04, and 1.01E-05 for females, respectively. Hence, Table 6 shows that soils of the study regions had HQ_Dermal_ values for trace elements below 1.0, indicating that trace metal levels in soils in the Jamalpur district study regions were within an acceptable range of non-carcinogenic detrimental human health concerns.

**Table 6:** Calculated chronic daily intake (CDI), hazard quotients (HQ) and incremental lifetime cancer risk (ILCR) values for male and female due to dermal exposure of trace metals to the soils of brinjal-producing areas of Jamalpur district, Bangladesh.

Metals	CDI_Dermal_						HQ_Dermal_						ILCR_Dermal_					
	Male			Female			Male			Female			Male			Female		
	Min	Max	Mean	Min	Max	Mean	Min	Max	Mean	Min	Max	Mean	Min	Max	Mean	Min	Max	Mean
Pb	4.97E-08	1.29E-07	9.25E-08	6.96E-08	1.81E-07	1.30E-07	4.61E-05	1.20E-04	8.57E-05	6.45E-05	1.68E-04	1.20E-04	6.03E-10	1.57E-09	1.12E-09	8.44E-10	2.20E-09	1.57E-09
Ni	7.68E-08	1.37E-08	1.14E-08	1.08E-08	1.92E-08	1.59E-08	E9.60–05	1.71E-04	1.42E-04	1.34E-04	2.40E-04	1.99E-04	7.49E-07	**1.34E-06**	**1.11E-06**	**1.05E-06**	**1.87E-06**	**1.56E-06**
Cd	0.00E + 00	3.64E-09	1.76E-09	0.00E + 00	5.10E-09	2.47E-09	0.00E + 00	1.46E-04	7.05E-05	0.00E + 00	2.04E-04	9.87E-05	0.00E + 00	9.36E-07	4.53E-07	0.00E + 00	**1.31E-06**	6.34E-07
Cu	1.18E-07	2.67E-07	1.74E-07	1.66E-07	3.73E-07	2.43E-07	9.87E-06	2.22E-05	1.45E-05	1.38E-05	3.11E-05	2.03E-05	–	–	–	–	–	–
Cr	2.50E-07	4.11E-07	3.42E-07	3.50E-07	5.76E-07	4.79E-07	1.92E-02	3.17E-02	2.63E-02	2.69E-02	4.43E-02	3.69E-02	–	–	–	–	–	–
Fe	1.48E-04	1.98E-04	1.74E-04	2.08E-04	2.78E-04	2.44E-04	3.53E-04	4.72E-04	4.15E-04	4.94E-04	6.61E-04	5.81E-04	–	–	–	–	–	–
Mn	2.22E-06	3.30E-06	2.58E-06	3.11E-06	4.62E-06	3.61E-06	3.97E-04	5.89E-04	4.60E-04	5.56E-04	8.25E-04	6.44E-04	–	–	–	–	–	–
Zn	3.69E-07	5.76E-07	4.34E-07	5.16E-07	8.07E-07	6.08E-07	6.15E-06	9.60E-06	7.24E-06	8.61E-06	1.34E-05	1.01E-05	–	–	–	–	–	–

Bold ILCR_Dermal_ values indicates the cancer risk.

The mean calculated CDI_Oral_ values for Pb, Ni, Cd, Cu, Cr, Fe, Mn and Zn were 0.045, 0.012, 0.002, 0.228, 0.000, 0.486, 0.024, and 0.279 mg kg^-1^ day^-1^ for males, and 0.063, 0.017, 0.003, 0.319, 0.000, 0.680, 0.034, and 0.391 mg kg^-1^ day^-1^ for females, respectively (Table 7). The average calculated HQ_Oral_ values for Pb, Ni, Cd, Cu, Cr, Fe, Mn, and Zn were 12.81, 0.60, 1.88, 5.69, 0.00, 0.69, 0.17, and 0.93 for males, and 17.94, 0.84, 2.64, 7.97, 0.00, 0.97, 0.24, and 1.30 for females, respectively (Table 7). The study results revealed non-carcinogenic risks (HQ_Oral_) of Pb and Cu for both males and females (HQ_Oral_ > 1.00) due to dietary intake of all samples of the study area. Similarly, the calculated HQ_Oral_ of Zn for females in all samples also had values > 1.00, thus hazardous for a human being. Furthermore, it can be summarized from the study that the calculated HQ_Oral_ of Cd in 40% of farmers′ field samples had HQ_Oral_ values > 1.00 for both males and females and Ni in 35% and Fe in 50% samples possessed values > 1.00 for females only, thus harmful for a human being (Table 9 Suppl.). On the other hand, the calculated HQ_Oral_ value < 1.00 means trace metal contents in those samples were below the non-carcinogenic risk threshold. Islam et al.^61^ also reported non-carcinogenic potential health risks of trace metals (Cd, Pb, Cr, and As) due to the consumption of vegetables. Almost similar observations were also reported by Islam et al.^17^. They stated that the HQ of trace metals through the dietary intake of vegetables decreased in the order of Cd > Cu > As > Pb > Ni > Cr.

**Table 7:** Calculated chronic daily intake (CDI), hazard quotients (HQ) and incremental lifetime cancer risk (ILCR) values for male and female due to oral exposure of trace metals from the dietary intake of brinjal fruits collected from different producer levels.

Metals	CDI_Oral_						HQ_Oral_						ILCR_Oral_					
	Male			Female			Male			Female			Male			Female		
	Min	Max	Mean	Min	Max	Mean	Min	Max	Mean	Min	Max	Mean	Min	Max	Mean	Min	Max	Mean
Pb	0.021	0.076	0.045	0.030	0.106	0.063	*6.06*	*21.67*	*12.81*	*8.48*	*30.34*	*17.94*	**1.80E-04**	**6.45E-04**	**3.81E-04**	**2.52E-04**	**9.03E-04**	**5.34E-04**
Ni	0.003	0.022	0.012	0.005	0.031	0.017	0.16	*1.10*	0.60	0.23	*1.55*	0.84	**2.94E-03**	**2.01E-02**	**1.09E-02**	**4.12E-03**	**2.81E-02**	**1.52E-02**
Cd	0.000	0.006	0.002	0.000	0.009	0.003	0.00	*6.36*	*1.88*	0.00	*8.90*	*2.64*	0.00E + 00	**9.54E-02**	**2.83E-02**	0.00E + 00	**1.34E-01**	**3.96E-02**
Cu	0.189	0.277	0.228	0.265	0.388	0.319	*4.73*	*6.94*	*5.69*	*6.62*	*9.71*	*7.97*	–	–	–	–	–	–
Cr	0.000	0.000	0.000	0.000	0.000	0.000	0.00	0.00	0.00	0.00	0.00	0.00	–	–	–	–	–	–
Fe	0.340	0.615	0.486	0.476	0.860	0.680	0.49	0.88	0.69	0.68	*1.23*	0.97	–	–	–	–	–	–
Mn	0.000	0.090	0.024	0.000	0.126	0.034	0.00	0.64	0.17	0.00	0.90	0.24	–	–	–	–	–	–
Zn	0.225	0.400	0.279	0.314	0.560	0.391	0.75	*1.33*	0.93	*1.05*	*1.87*	*1.30*	–	–	–	–	–	–

Italic HQ_Oral_ values indicate non-cancer risk and bold ILCR_Oral_ values indicate the cancer risk.

#### Carcinogenic health risk

Toxic metals (viz. Pb, Cd, and Ni) have long been known to be carcinogenic to humans. According to Kim et al.^67^, Cd and Ni are classified as a group 1 carcinogen in humans, while Pb is classified as a group 2A probable human carcinogen by the International Agency for Research on Cancer (IARC). Toxic metals cause oxidative stress, altered gene expression, and cell death, all of which raise the risk of carcinoma and melanoma disorders^67^. The calculated incremental lifetime cancer risks (ILCR_Dermal_) posed by Pb, Ni, and Cd due to dermal exposure to the topsoils of the study regions varied from 6.03E-10 to 1.57E-09, 7.49E-07 to 1.34E-06, and 0.00E + 00 to 9.36E-07 for males and 8.44E-10 to 2.20E-09, 1.05E-06 to 1.87E-06, and 0.00E + 00 to 1.31E-06 for females, respectively (Table 6). Carcinogenic health risks of less than one chance in 1.00E-06 are regarded as minimal, and a value of 1.00E-06 to 1.00E-04 is considered tolerable^23^. However, the calculated ILCR_Dermal_ values for Ni (65 and 100% samples for males and females, respectively) and Cd (50% samples for females only) showed values within the acceptable range of carcinogenic risk index as proposed by the USEPA, while others were smaller than this range. Hence, the present study summarized that the risk of developing cancer due to dermal absorption of toxic metals in soils in the study regions could be considered negligible (Tables 6 & 7 Suppl.).

The calculated ILCR_Oral_ values for Pb, Ni, and Cd due to ingestion exposure of brinjal fruits collected from farmer′s field are presented in Table 7. The values ranged from 1.80E-04 to 6.45E-04, 2.94E-03 to 2.01E-02, and 0.00E+00 to 9.54E-02 with the mean values of 3.81E-04, 1.09E-02, and 2.83E-02 for males, and 2.52E-04 to 9.03E-04, 4.12E-03 to 2.81E-02, and 0.00E+00 to 1.34E-01 with the mean values of 5.34E-04, 1.52E-02, and 3.96E-02 for females, respectively. Thus, this study revealed that the calculated ILCR_Oral_ values for Pb and Ni in all samples and Cd in 40% of samples were several hundred times higher for males and females than the threshold (1.00 × 10^–6^ to 1.00 × 10^–4^) (Tables 7 & 10 Suppl.). Such high ILCR values suggested that consumers in the country who ate brinjal grown in the study area of Jamalpur, Bangladesh, were at much higher cancer risks. Islam et al.^61^ also reported that the probable health threat to people of Bangladesh from As and Pb exposure from the dietary intake of vegetables should not be overlooked, and the residents are susceptible to carcinogenic risks. As mentioned earlier, the application of various synthetic substances, irrigation water, fertilizers, and pesticides, might be a source of these toxic metals in brinjal fruits, which is at par with the observations of Ahmad and Goni^55^. Furthermore, they also concluded that long-term intake of such metal-contaminated vegetables could promote thalassemia, dermatitis, brain and kidney damage, and even cancer in humans.

### Principal component analysis (PCA)

Principal Component Analysis (PCA) allows us to deduce how certain variables characterize the target substances and define their associations^68^. PCA also calculates the structural relationship of the data by identifying additional hypothetical variables (principal components, PC) that account for as much variation (or correlation) as feasible in a multi-dimensional data set. This approach aids in the identification of groupings of variables (for example, trace metals in vegetables and soil) based on weight and sample classes based on scores^69^.

**Figure 4:**
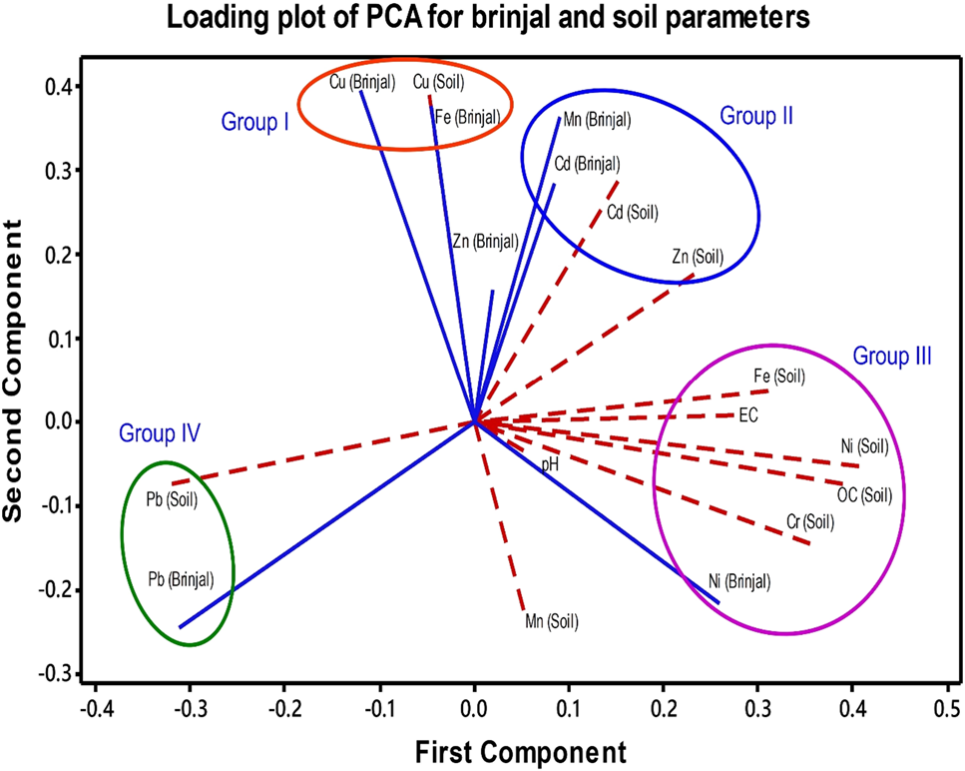
Loading plot presentation of the Principal Component Analysis (PCA) results showing the brinjal fruit (blue lines) and soil (dashed red lines) parameters. In the figure, the length of each eigenvector is proportional to the variance in the data for that variable. The angle between the eigenvectors represents the correlations among the different variables. The colored circle groups of variables indicated by I, II, III and IV show strong positive correlations with each other.

The PCA was used to determine the trace metal content of brinjal and soil. Figure 4 and Table 11 (Suppl.) show the loading plot of the PCA findings for various variables, as well as their Eigen analysis of data. In Fig. 4, the length of each eigenvector is proportionate to the variation in the data for independent factors, and the angle between the eigenvectors denotes the correlations between the soil and brinjal variables. In the figure, the colored circle sets of soil and brinjal characteristics represented by I, II, III, and IV demonstrated substantial positive associations. Strong positive correlations were observed for soil and brinjal Cu (group I), Cd and Zn (group II), Ni (group III), and Pb (group IV). Such a positive association is an indication that these metals accumulate in brinjal fruits from the soil. On the other hand, however, Fe and Mn in brinjal do not correspond with the soil level. Interestingly, soil OC, pH and EC were strongly correlated with soil Fe, Cr and Ni contents. However, only Ni content was synchronized in both soil and brinjal fruits (group III).

Usually, Cd and Zn showed antagonistic behaviour for plant uptake. However, higher amounts of Zn and Cd as impurities have been reported in phosphatic fertilizers^62^, and commercial and regularly sold Zn fertilizers have also been discovered to have large quantities of Cd contaminants^70^. In this study, soil Cd level showed a positive correlation with soil Zn (group II). Metals like Ni, Fe, and Cr in soil are closely correlated with soil organic carbon (OC), and possibly, soil organic matter may be a major contributor to the release of these trace elements in the soil. The PCA results suggest that the content of trace metals in soils is an important source for those elements (viz. Pb, Cd, Ni, and Cu) in brinjal. Thus, a further pinpoint investigation should be designed to limit the toxic elements in all kinds of used agricultural inputs, particularly organic and inorganic fertilizers, irrigation water, and pesticides, to ensure soil quality and safe agricultural production.

## Conclusions

Contamination of foodstuffs by different toxic trace elements is alarming worldwide, including in Bangladesh. Our current study examined the total trace metal content of topsoils and brinjal fruits harvested from a brinjal-producing hotspot in Bangladesh i.e. Jamalpur district. The study's findings suggested that using various synthetic materials, such as inorganic fertilizers and pesticides, along with manures and irrigation water, could become a cause of toxic elements in brinjal fruits, which would need to be confirmed by the extensive investigation. Thus, monitoring trace metals in vegetables and other aspects of nature on a routine basis is critical for identifying sources of contamination and preventing or reducing crop (and human) exposure to excessive levels of these pollutants. Moreover, our study results also suggested that toxic metals deposited in soils are an important source for those elements accumulated in brinjal. Therefore, this uncertain entry point for toxic elements into the vegetable supply chain should indeed be considered a serious roadblock to Bangladesh′s food safety. Furthermore, a thorough evaluation should also be conducted to confirm the level of trace elements in other vegetables and grains that could accumulate hazardous elements at a faster rate than brinjal.

## Supplementary Information

Supplementary Information.

## Data Availability

The data that support the findings of this study are available on request from the corresponding author.
