# Supplementary material for: Human health implications of trace metal contamination in topsoils and brinjal fruits harvested from a famous brinjal-producing area in Bangladesh
Source: Sci Rep. 2022 Aug 22;12:14278. doi: 10.1038/s41598-022-17930-5 (PMC9395429; doi:10.1038/s41598-022-17930-5)
Supplement: Supplementary file 1 — Supplementary Information. [file 41598_2022_17930_MOESM1_ESM.docx]

**Title:** Human health implications of trace metal contamination in topsoils and brinjal fruits harvested from a famous brinjal-producing area in Bangladesh

**Names of authors:** Anika Bushra^1^, H. M. Zakir^1^*, Shaila Sharmin^2^, Q. F. Quadir^1^, M. H. Rashid^3^, M. S. Rahman^1^ and Supti Mallick^1^

**Affiliation and address of authors-**

^1^Laboratory of Plant Nutrition and Environmental Chemistry, Department of Agricultural Chemistry, Faculty of Agriculture, Bangladesh Agricultural University, Mymensingh-2202, Bangladesh. E-mails: [bushraanika642@gmail.com](mailto:bushraanika642@gmail.com); [zakirhm_ac@bau.edu.bd](mailto:zakirhm_ac@bau.edu.bd); [qfq@bau.edu.bd](mailto:qfq@bau.edu.bd); supti.ac@bau.edu.bd; [shahinur.achem@bau.edu.bd](mailto:shahinur.achem@bau.edu.bd);

^2^College of Agricultural Sciences, International University of Business Agriculture and Technology (IUBAT), Uttara Model Town, Dhaka-1230, Bangladesh. E-mail: shaila.sharmin@iubat.edu

^3^Department of Agronomy, Faculty of Agriculture, Bangladesh Agricultural University, Mymensingh-2202, Bangladesh. Email: [mhrashid@bau.edu.bd](mailto:mhrashid@bau.edu.bd)

**The address, e-mail, telephone and fax numbers, and ORCID identifier of the corresponding author-**

Dr. Md. Zakir Hossen

Professor

Laboratory of Plant Nutrition and Environmental Chemistry, Department of Agricultural Chemistry, Faculty of Agriculture, Bangladesh Agricultural University, Mymensingh-2202, Bangladesh.

E-mail: [zakirhm.ac.bau@gmail.com](mailto:zakirhm.ac.bau@gmail.com); zakirhm_ac@bau.edu.bd

Telephone: +880-91-67401-06/65917 (off.); Mobile: +880-1733513640

Fax.: +880-91-61510

ORCID identifier: 0000-0002-2970-5042

**Table 1 (Suppl.): Details of operating parameters for determination of different trace metals using atomic absorption spectrophotometer (AAS)**

| **Trace metals** | **Analytical wavelength (nm)** | **Concentration limit of detection (µg g^-1^)** | **Concentration of standard series solution used in calibration (µg mL^-1^)** | **Obtained equation with R^2^ value** |
| --- | --- | --- | --- | --- |
| **Pb** | 217.0 | 0.01 | 0.0, 1.0, 2.0, 3.0, 4.0 and 5.0 | Y = 0.0175x – 0.0113; R^2^ = 0.998 |
| **Ni** | 232.0 | 0.01 | 0.0, 0.5, 1.0, 2.0 and 4.0 | Y = 0.0969x – 0.0065; R^2^ = 0.999 |
| **Cd** | 228.8 | <0.01 ̴ 0.09 | 0.0, 0.1, 0.2, 0.4 and 0.8 | Y = 0.5669x + 0.0043; R^2^ = 0.999 |
| **Cr** | 357.9 | 0.01 | 0.0, 0.5, 1.0, 2.0 and 4.0 | Y = 0.0986x – 0.0143; R^2^ = 0.999 |
| **Cu** | 324.7 | <0.01 ̴ 0.09 | 0.0, 0.5, 1.0, 2.0 and 4.0 | Y = 0.1235x + 0.0251; R^2^ = 0.999 |
| **Fe** | 271.9 | 0.01 | 0.0, 1.0, 2.0, 4.0 and 8.0 | Y = 0.0922x + 0.0007; R^2^ = 0.999 |
| **Mn** | 279.5 | 0.01 | 0.0, 1.0, 3.0, 5.0 and 7.0 | Y = 0.1850x + 0.0345; R^2^ = 0.999 |
| **Zn** | 213.9 | <0.01 ̴ 0.09 | 0.0, 0.1, 0.2, 0.3, 0.4 and 0.5 | Y = 0.4542x + 0.0019; R^2^ = 0.999 |

**Table 2 (Suppl.): Comparative analysis of different trace metal contents (in µg g^-1^) in soils of brinjal-producing areas of Jamalpur, Bangladesh with geochemical background and toxicological reference values**

|  | **Metal contents (in µg g^-1^)** | | | | | | | |
| --- | --- | --- | --- | --- | --- | --- | --- | --- |
|  | **Pb** | **Ni** | **Cd** | **Cr** | **Cu** | **Fe** | **Mn** | **Zn** |
| Present study average | 16.9 | 20.8 | 0.32 | 62.6 | 31.8 | 31882 | 471.6 | 79.4 |
| Average shale value ^a^ | 20 | 68 | 0.30 | 90 | 45 | 47200 | 850 | 95 |
| Crustal average ^b^ | 12.5 | 75 | 0.20 | 100 | 55 | 56300 | 950 | 70 |
| Earth’s crust (upper continental crust) ^c^ | 16 | 58 | 0.13 | 83 | 47 | 46500 | 1000 | 83 |
| USEPA toxicity reference value (TRV) ^d^ | 100 | 100 | 10.00 | - | 32 | - | - | 199 |
| Canadian soil quality guideline ^e^ | 70 | 50 | 1.40 | 64 | 63 | - | - | 200 |
| Soil quality guidelines- Netherlands ^f^ | 85 | 35 | 0.80 | 100 | 36 | - | - | 140 |

^a^ = Turekian and Wedepohl [47]; ^b^ = Taylor [21]; ^c^ = Yaroshevsky [51]; ^d^ = USEPA [48]; ^e^ = CCME [49]; ^f^ = Swartjes et al. [50].

**Table 3 (Suppl.): Trace metal concentrations in soil samples collected from different brinjal cultivating areas of Melandaha and Islampur Upazila of Jamalpur district, Bangladesh.**

| **Name of Upazila** | **Sample ID** | **Metal contents in μg g^-1^ (Mean±SD)** | | | | | | | |
| --- | --- | --- | --- | --- | --- | --- | --- | --- | --- |
|  |  | **Pb** | **Ni** | **Cd** | **Cu** | **Cr** | **Fe** | **Mn** | **Zn** |
| Melandaha | 1 | 16.46±3.30 | 22.39±0.41 | 0.564±0.15 | 46.73±0.72 | 65.64±0.86 | 36262±190 | 451.3±2.27 | 105.42±0.71 |
|  | 2 | 17.22±0.90 | 24.55±0.25 | 0.664±0.16 | 21.67±0.71 | 72.54±0.76 | 36304±147 | 406.6±2.48 | 73.50±0.59 |
|  | 3 | 10.18±2.86 | 21.39±0.16 | 0.547±0.04 | 39.99±0.79 | 61.88±0.12 | 34386±78 | 446.8±1.50 | 83.01±0.51 |
|  | 4 | 16.11±2.68 | 18.41±0.14 | 0.649±0.01 | 32.04±0.76 | 53.08±0.11 | 31118±365 | 412.5±0.76 | 69.73±0.76 |
|  | 5 | 17.94±1.72 | 20.62±0.21 | 0.506±0.22 | 34.92±0.50 | 71.91±1.07 | 35170±189 | 526.8±1.32 | 67.81±0.03 |
|  | 6 | 14.77±1.52 | 23.22±0.11 | 0.556±0.25 | 48.79±0.70 | 63.34±0.66 | 33865±417 | 433.6±3.66 | 90.19±0.61 |
|  | 7 | 22.48±4.50 | 16.70±0.38 | <0.010±0.00 | 28.11±0.18 | 52.36±0.38 | 31197±144 | 551.5±2.73 | 75.35±0.26 |
|  | 8 | 21.14±1.50 | 14.05±0.24 | <0.010±0.00 | 45.93±0.54 | 45.76±0.38 | 27126±330 | 423.9±0.66 | 67.49±0.29 |
|  | 9 | 23.66±4.47 | 17.25±0.54 | <0.010±0.00 | 36.72±0.33 | 55.90±0.62 | 29669±81 | 416.9±2.74 | 70.35±0.35 |
| Islampur | 10 | 21.14±1.56 | 18.74±0.22 | <0.010±0.00 | 26.67±0.54 | 56.48±0.74 | 30671±216 | 441.5±3.78 | 77.48±0.57 |
|  | 11 | 23.17±3.76 | 18.59±0.18 | 0.666±0.12 | 25.34±0.63 | 53.89±0.58 | 27511±348 | 471.8±2.93 | 83.46±1.77 |
|  | 12 | 19.61±1.33 | 17.66±0.30 | 0.541±0.05 | 26.43±0.62 | 71.31±0.10 | 29518±399 | 523.4±2.56 | 73.73±0.74 |
|  | 13 | 20.15±1.18 | 23.49±0.26 | <0.010±0.00 | 25.48±0.46 | 75.28±0.52 | 35245±511 | 471.9±3.18 | 83.02±0.59 |
|  | 14 | 16.24±2.03 | 20.86±0.29 | 0.543±0.07 | 24.50±0.22 | 62.01±0.43 | 29428±423 | 447.8±2.33 | 81.14±0.12 |
|  | 15 | 15.79±0.91 | 22.08±0.77 | <0.010±0.00 | 28.39±0.50 | 66.21±0.34 | 33758±360 | 422.4±0.46 | 81.72±0.71 |
|  | 16 | 11.97±1.37 | 25.08±0.16 | 0.632±0.07 | 33.05±0.43 | 53.08±0.11 | 27975±392 | 482.3±1.63 | 83.11±0.86 |
|  | 17 | 12.60±3.79 | 23.69±0.60 | <0.010±0.00 | 29.06±0.37 | 69.79±0.88 | 34094±243 | 493.9±2.88 | 82.14±0.41 |
|  | 18 | 18.66±3.10 | 21.95±0.58 | <0.010±0.00 | 26.16±0.63 | 63.99±0.44 | 31140±40 | 604.0±3.18 | 80.80±0.69 |
|  | 19 | 10.11±2.41 | 22.90±0.06 | 0.580±0.07 | 26.73±0.38 | 70.55±0.67 | 33843±244 | 494.2±4.48 | 82.37±0.50 |
|  | 20 | 9.10±0.58 | 23.25±0.44 | <0.010±0.00 | 29.46±0.80 | 67.53±0.63 | 29368±301 | 508.0±0.71 | 77.08±0.36 |
| **Average** | | **16.93** | **20.84** | **0.322** | **31.81** | **62.63** | **31882** | **471.6** | **79.44** |
| **Minimum** | | **9.10** | **14.05** | **<0.010** | **21.67** | **45.76** | **27126** | **406.6** | **67.49** |
| **Maximum** | | **23.66** | **25.08** | **0.666** | **48.79** | **75.28** | **36304** | **604.0** | **105.42** |

SD means standard deviation.

**Table 4 (Suppl.): Trace metal concentrations in brinjal fruits collected from farmers′ fields of Melandaha and Islampur Upazila of Jamalpur district, Bangladesh.**

| **Name of Upazila** | **Sample ID** | **Metal contents in μg g^-1^ (Mean±SD)** | | | | | | | |
| --- | --- | --- | --- | --- | --- | --- | --- | --- | --- |
|  |  | **Pb** | **Ni** | **Cd** | **Cu** | **Cr** | **Fe** | **Mn** | **Zn** |
| Melandaha | 1 | 0.204±0.25 | 0.097±0.053 | 0.053±0.008 | 2.52±0.023 | <0.01±0.000 | 5.33±0.009 | 0.820±0.003 | 2.81±0.011 |
|  | 2 | 0.287±0.14 | 0.109±0.031 | 0.041±0.010 | 1.82±0.016 | <0.01±0.000 | 5.20±0.084 | 0.084±0.014 | 2.33±0.011 |
|  | 3 | 0.337±0.20 | 0.069±0.022 | 0.044±0.005 | 2.50±0.050 | <0.01±0.000 | 5.91±0.073 | 0.866±0.071 | 2.72±0.016 |
|  | 4 | 0.388±025 | 0.031±0.014 | 0.047±0.012 | 2.05±0.015 | <0.01±0.000 | 4.68±0.081 | 0.130±0.012 | 2.28±0.010 |
|  | 5 | 0.278±0.16 | 0.099±0.048 | <0.01±0.000 | 2.40±0.022 | <0.01±0.000 | 4.74±0.044 | 0.267±0.023 | 2.59±0.015 |
|  | 6 | 0.312±0.28 | 0.138±0.047 | <0.01±0.000 | 2.67±0.033 | <0.01±0.000 | 5.17±0.099 | 0.516±0.023 | 3.30±0.039 |
|  | 7 | 0.729±0.12 | 0.092±0.070 | <0.01±0.000 | 2.06±0.030 | <0.01±0.000 | 5.01±0.018 | 0.240±0.031 | 2.45±0.022 |
|  | 8 | 0.536±0.25 | 0.049±0.029 | 0.040±0.019 | 2.56±0.033 | <0.01±0.000 | 5.04±0.035 | 0.172±0.009 | 2.54±0.021 |
|  | 9 | 0.673±0.21 | 0.093±0.021 | <0.01±0.000 | 2.42±0.028 | <0.01±0.000 | 4.24±0.144 | 0.047±0.025 | 2.60±0.023 |
| Islampur | 10 | 0.633±0.17 | 0.127±0.048 | <0.01±0.000 | 2.21±0.044 | <0.01±0.000 | 4.46±0.068 | 0.361±0.019 | 2.48±0.022 |
|  | 11 | 0.477±0.14 | 0.078±0.032 | 0.033±0.006 | 2.28±0.027 | <0.01±0.000 | 5.23±0.063 | 0.019±0.011 | 3.30±0.013 |
|  | 12 | 0.442±0.12 | 0.094±0.004 | <0.01±0.000 | 2.23±0.029 | <0.01±0.000 | 5.75±0.065 | 0.479±0.034 | 2.84±0.012 |
|  | 13 | 0.660±0.07 | 0.150±0.021 | <0.01±0.000 | 2.00±0.038 | <0.01±0.000 | 4.07±0.119 | <0.01±0.000 | 3.85±0.023 |
|  | 14 | 0.399±0.09 | 0.108±0.034 | <0.01±0.000 | 2.02±0.038 | <0.01±0.000 | 3.91±0.066 | <0.01±0.000 | 2.49±0.016 |
|  | 15 | 0.341±0.03 | 0.129±0.035 | <0.01±0.000 | 2.00±0.003 | <0.01±0.000 | 4.15±0.098 | <0.01±0.000 | 2.34±0.040 |
|  | 16 | 0.246±0.05 | 0.182±0.050 | 0.043±0.009 | 2.12±0.025 | <0.01±0.000 | 5.17±0.018 | 0.077±0.055 | 3.64±0.027 |
|  | 17 | 0.490±0.16 | 0.149±0.052 | <0.01±0.000 | 1.87±0.035 | <0.01±0.000 | 3.77±0.113 | 0.021±0.035 | 2.16±0.008 |
|  | 18 | 0.510±0.11 | 0.143±0.052 | <0.01±0.000 | 1.89±0.031 | <0.01±0.000 | 3.27±0.066 | 0.156±0.008 | 2.17±0.003 |
|  | 19 | 0.295±0.25 | 0.150±0.051 | 0.061±0.007 | 2.01±0.009 | <0.01±0.000 | 3.43±0.013 | 0.069±0.021 | 2.24±0.004 |
|  | 20 | 0.387±0.12 | 0.212±0.074 | <0.01±0.000 | 2.14±0.035 | <0.01±0.000 | 4.93±0.055 | 0.291±0.024 | 2.56±0.009 |
| **Average** | | **0.431** | **0.115** | **0.018** | **2.19** | **<0.01** | **4.67** | **0.231** | **2.69** |
| **Minimum** | | **0.204** | **0.031** | **<0.01** | **1.82** | **<0.01** | **3.27** | **<0.01** | **2.16** |
| **Maximum** | | **0.729** | **0.212** | **0.061** | **2.67** | **<0.01** | **5.91** | **0.866** | **3.85** |

SD means standard deviation.

**Table 5 (Suppl.):** **Calculated non-carcinogenic chronic daily intake (CDI_Dermal_) values for male and female due to dermal exposure of trace metals to the soils of brinjal-producing areas of Jamalpur district, Bangladesh**

| **Name of Upazila** | **Sample ID** | **Pb** | | **Ni** | | **Cd** | | **Cr** | | **Cu** | | **Fe** | | **Mn** | | **Zn** | |
| --- | --- | --- | --- | --- | --- | --- | --- | --- | --- | --- | --- | --- | --- | --- | --- | --- | --- |
|  |  | **Male** | **Female** | **Male** | **Female** | **Male** | **Female** | **Male** | **Female** | **Male** | **Female** | **Male** | **Female** | **Male** | **Female** | **Male** | **Female** |
| Melandaha | 1 | 9.00E-08 | 1.26E-07 | 1.22E-07 | 1.71E-07 | 3.08E-09 | 4.32E-09 | 3.59E-07 | 5.02E-07 | 2.55E-07 | 3.58E-07 | 1.98E-04 | 2.77E-04 | 2.47E-06 | 3.45E-06 | 5.76E-07 | 8.07E-07 |
|  | 2 | 9.41E-08 | 1.32E-07 | 1.34E-07 | 1.88E-07 | 3.63E-09 | 5.08E-09 | 3.96E-07 | 5.55E-07 | 1.18E-07 | 1.66E-07 | 1.98E-04 | 2.78E-04 | 2.22E-06 | 3.11E-06 | 4.02E-07 | 5.62E-07 |
|  | 3 | 5.56E-08 | 7.79E-08 | 1.17E-07 | 1.64E-07 | 2.99E-09 | 4.19E-09 | 3.38E-07 | 4.73E-07 | 2.19E-07 | 3.06E-07 | 1.88E-04 | 2.63E-04 | 2.44E-06 | 3.42E-06 | 4.54E-07 | 6.35E-07 |
|  | 4 | 8.80E-08 | 1.23E-07 | 1.01E-07 | 1.41E-07 | 3.55E-09 | 4.96E-09 | 2.90E-07 | 4.06E-07 | 1.75E-07 | 2.45E-07 | 1.70E-04 | 2.38E-04 | 2.25E-06 | 3.16E-06 | 3.81E-07 | 5.34E-07 |
|  | 5 | 9.81E-08 | 1.37E-07 | 1.13E-07 | 1.58E-07 | 2.77E-09 | 3.87E-09 | 3.93E-07 | 5.50E-07 | 1.91E-07 | 2.67E-07 | 1.92E-04 | 2.69E-04 | 2.88E-06 | 4.03E-06 | 3.71E-07 | 5.19E-07 |
|  | 6 | 8.08E-08 | 1.13E-07 | 1.27E-07 | 1.78E-07 | 3.04E-09 | 4.26E-09 | 3.46E-07 | 4.85E-07 | 2.67E-07 | 3.73E-07 | 1.85E-04 | 2.59E-04 | 2.37E-06 | 3.32E-06 | 4.93E-07 | 6.90E-07 |
|  | 7 | 1.23E-07 | 1.72E-07 | 9.13E-08 | 1.28E-07 | 0.00E+00 | 0.00E+00 | 2.86E-07 | 4.01E-07 | 1.54E-07 | 2.15E-07 | 1.71E-04 | 2.39E-04 | 3.01E-06 | 4.22E-06 | 4.12E-07 | 5.77E-07 |
|  | 8 | 1.16E-07 | 1.62E-07 | 7.68E-08 | 1.08E-07 | 0.00E+00 | 0.00E+00 | 2.50E-07 | 3.50E-07 | 2.51E-07 | 3.51E-07 | 1.48E-04 | 2.08E-04 | 2.32E-06 | 3.24E-06 | 3.69E-07 | 5.16E-07 |
|  | 9 | 1.29E-07 | 1.81E-07 | 9.43E-08 | 1.32E-07 | 0.00E+00 | 0.00E+00 | 3.06E-07 | 4.28E-07 | 2.01E-07 | 2.81E-07 | 1.62E-04 | 2.27E-04 | 2.28E-06 | 3.19E-06 | 3.85E-07 | 5.38E-07 |
| Islampur | 10 | 1.16E-07 | 1.62E-07 | 1.02E-07 | 1.43E-07 | 0.00E+00 | 0.00E+00 | 3.09E-07 | 4.32E-07 | 1.46E-07 | 2.04E-07 | 1.68E-04 | 2.35E-04 | 2.41E-06 | 3.38E-06 | 4.23E-07 | 5.93E-07 |
|  | 11 | 1.27E-07 | 1.77E-07 | 1.02E-07 | 1.42E-07 | 3.64E-09 | 5.10E-09 | 2.95E-07 | 4.12E-07 | 1.38E-07 | 1.94E-07 | 1.50E-04 | 2.11E-04 | 2.58E-06 | 3.61E-06 | 4.56E-07 | 6.39E-07 |
|  | 12 | 1.07E-07 | 1.50E-07 | 9.65E-08 | 1.35E-07 | 2.95E-09 | 4.14E-09 | 3.90E-07 | 5.46E-07 | 1.44E-07 | 2.02E-07 | 1.61E-04 | 2.26E-04 | 2.86E-06 | 4.00E-06 | 4.03E-07 | 5.64E-07 |
|  | 13 | 1.10E-07 | 1.54E-07 | 1.28E-07 | 1.80E-07 | 0.00E+00 | 0.00E+00 | 4.11E-07 | 5.76E-07 | 1.39E-07 | 1.95E-07 | 1.93E-04 | 2.70E-04 | 2.58E-06 | 3.61E-06 | 4.54E-07 | 6.35E-07 |
|  | 14 | 8.88E-08 | 1.24E-07 | 1.14E-07 | 1.60E-07 | 2.97E-09 | 4.16E-09 | 3.39E-07 | 4.74E-07 | 1.34E-07 | 1.87E-07 | 1.61E-04 | 2.25E-04 | 2.45E-06 | 3.43E-06 | 4.43E-07 | 6.21E-07 |
|  | 15 | 8.63E-08 | 1.21E-07 | 1.21E-07 | 1.69E-07 | 0.00E+00 | 0.00E+00 | 3.62E-07 | 5.07E-07 | 1.55E-07 | 2.17E-07 | 1.85E-04 | 2.58E-04 | 2.31E-06 | 3.23E-06 | 4.47E-07 | 6.25E-07 |
|  | 16 | 6.54E-08 | 9.16E-08 | 1.37E-07 | 1.92E-07 | 3.45E-09 | 4.84E-09 | 2.90E-07 | 4.06E-07 | 1.81E-07 | 2.53E-07 | 1.53E-04 | 2.14E-04 | 2.64E-06 | 3.69E-06 | 4.54E-07 | 6.36E-07 |
|  | 17 | 6.89E-08 | 9.64E-08 | 1.29E-07 | 1.81E-07 | 0.00E+00 | 0.00E+00 | 3.81E-07 | 5.34E-07 | 1.59E-07 | 2.22E-07 | 1.86E-04 | 2.61E-04 | 2.70E-06 | 3.78E-06 | 4.49E-07 | 6.29E-07 |
|  | 18 | 1.02E-07 | 1.43E-07 | 1.20E-07 | 1.68E-07 | 0.00E+00 | 0.00E+00 | 3.50E-07 | 4.90E-07 | 1.43E-07 | 2.00E-07 | 1.70E-04 | 2.38E-04 | 3.30E-06 | 4.62E-06 | 4.42E-07 | 6.18E-07 |
|  | 19 | 5.53E-08 | 7.74E-08 | 1.25E-07 | 1.75E-07 | 3.17E-09 | 4.44E-09 | 3.86E-07 | 5.40E-07 | 1.46E-07 | 2.05E-07 | 1.85E-04 | 2.59E-04 | 2.70E-06 | 3.78E-06 | 4.50E-07 | 6.30E-07 |
|  | 20 | 4.97E-08 | 6.96E-08 | 1.27E-07 | 1.78E-07 | 0.00E+00 | 0.00E+00 | 3.69E-07 | 5.17E-07 | 1.61E-07 | 2.25E-07 | 1.61E-04 | 2.25E-04 | 2.78E-06 | 3.89E-06 | 4.21E-07 | 5.90E-07 |
| **Min.** | | **4.97E-08** | **6.96E-08** | **7.68E-08** | **1.08E-07** | **0.00E+00** | **0.00E+00** | **2.50E-07** | **3.50E-07** | **1.18E-07** | **1.66E-07** | **1.48E-04** | **2.08E-04** | **2.22E-06** | **3.11E-06** | **3.69E-07** | **5.16E-07** |
| **Max.** | | **1.29E-07** | **1.81E-07** | **1.37E-07** | **1.92E-07** | **3.64E-09** | **5.10E-09** | **4.11E-07** | **5.76E-07** | **2.67E-07** | **3.73E-07** | **1.98E-04** | **2.78E-04** | **3.30E-06** | **4.62E-06** | **5.76E-07** | **8.07E-07** |
| **Mean** | | **9.25E-08** | **1.30E-07** | **1.14E-07** | **1.59E-07** | **1.76E-09** | **2.47E-09** | **3.42E-07** | **4.79E-07** | **1.74E-07** | **2.43E-07** | **1.74E-04** | **2.44E-04** | **2.58E-06** | **3.61E-06** | **4.34E-07** | **6.08E-07** |

**Table 6 (Suppl.):** **Calculated hazard quotient (HQ_Dermal_) values for male and female due to dermal exposure of trace metals to the soils of brinjal-producing areas of Jamalpur district, Bangladesh**

| **Name of Upazila** | **Sample ID** | **Pb** | | **Ni** | | **Cd** | | **Cr** | | **Cu** | | **Fe** | | **Mn** | | **Zn** | |
| --- | --- | --- | --- | --- | --- | --- | --- | --- | --- | --- | --- | --- | --- | --- | --- | --- | --- |
|  |  | **Male** | **Female** | **Male** | **Female** | **Male** | **Female** | **Male** | **Female** | **Male** | **Female** | **Male** | **Female** | **Male** | **Female** | **Male** | **Female** |
| Melandaha | 1 | 8.33E-05 | 1.17E-04 | 1.53E-04 | 2.14E-04 | 1.23E-04 | 1.73E-04 | 2.76E-02 | 3.86E-02 | 2.13E-05 | 2.98E-05 | 4.72E-04 | 6.61E-04 | 4.41E-04 | 6.17E-04 | 9.60E-06 | 1.34E-05 |
|  | 2 | 8.72E-05 | 1.22E-04 | 1.68E-04 | 2.35E-04 | 1.45E-04 | 2.03E-04 | 3.05E-02 | 4.27E-02 | 9.87E-06 | 1.38E-05 | 4.72E-04 | 6.61E-04 | 3.97E-04 | 5.56E-04 | 6.70E-06 | 9.37E-06 |
|  | 3 | 5.15E-05 | 7.21E-05 | 1.46E-04 | 2.05E-04 | 1.20E-04 | 1.67E-04 | 2.60E-02 | 3.64E-02 | 1.82E-05 | 2.55E-05 | 4.47E-04 | 6.26E-04 | 4.36E-04 | 6.11E-04 | 7.56E-06 | 1.06E-05 |
|  | 4 | 8.15E-05 | 1.14E-04 | 1.26E-04 | 1.76E-04 | 1.42E-04 | 1.99E-04 | 2.23E-02 | 3.12E-02 | 1.46E-05 | 2.04E-05 | 4.05E-04 | 5.67E-04 | 4.03E-04 | 5.64E-04 | 6.35E-06 | 8.89E-06 |
|  | 5 | 9.08E-05 | 1.27E-04 | 1.41E-04 | 1.97E-04 | 1.11E-04 | 1.55E-04 | 3.02E-02 | 4.23E-02 | 1.59E-05 | 2.23E-05 | 4.58E-04 | 6.41E-04 | 5.14E-04 | 7.20E-04 | 6.18E-06 | 8.65E-06 |
|  | 6 | 7.48E-05 | 1.05E-04 | 1.59E-04 | 2.22E-04 | 1.22E-04 | 1.70E-04 | 2.66E-02 | 3.73E-02 | 2.22E-05 | 3.11E-05 | 4.41E-04 | 6.17E-04 | 4.23E-04 | 5.93E-04 | 8.22E-06 | 1.15E-05 |
|  | 7 | 1.14E-04 | 1.59E-04 | 1.14E-04 | 1.60E-04 | 0.00E+00 | 0.00E+00 | 2.20E-02 | 3.08E-02 | 1.28E-05 | 1.79E-05 | 4.06E-04 | 5.68E-04 | 5.38E-04 | 7.54E-04 | 6.86E-06 | 9.61E-06 |
|  | 8 | 1.07E-04 | 1.50E-04 | 9.60E-05 | 1.34E-04 | 0.00E+00 | 0.00E+00 | 1.92E-02 | 2.69E-02 | 2.09E-05 | 2.93E-05 | 3.53E-04 | 4.94E-04 | 4.14E-04 | 5.79E-04 | 6.15E-06 | 8.61E-06 |
|  | 9 | 1.20E-04 | 1.68E-04 | 1.18E-04 | 1.65E-04 | 0.00E+00 | 0.00E+00 | 2.35E-02 | 3.29E-02 | 1.67E-05 | 2.34E-05 | 3.86E-04 | 5.41E-04 | 4.07E-04 | 5.70E-04 | 6.41E-06 | 8.97E-06 |
| Islampur | 10 | 1.07E-04 | 1.50E-04 | 1.28E-04 | 1.79E-04 | 0.00E+00 | 0.00E+00 | 2.37E-02 | 3.32E-02 | 1.21E-05 | 1.70E-05 | 3.99E-04 | 5.59E-04 | 4.31E-04 | 6.03E-04 | 7.06E-06 | 9.88E-06 |
|  | 11 | 1.17E-04 | 1.64E-04 | 1.27E-04 | 1.78E-04 | 1.46E-04 | 2.04E-04 | 2.27E-02 | 3.17E-02 | 1.15E-05 | 1.62E-05 | 3.58E-04 | 5.01E-04 | 4.61E-04 | 6.45E-04 | 7.60E-06 | 1.06E-05 |
|  | 12 | 9.92E-05 | 1.39E-04 | 1.21E-04 | 1.69E-04 | 1.18E-04 | 1.65E-04 | 3.00E-02 | 4.20E-02 | 1.20E-05 | 1.69E-05 | 3.84E-04 | 5.38E-04 | 5.11E-04 | 7.15E-04 | 6.72E-06 | 9.40E-06 |
|  | 13 | 1.02E-04 | 1.43E-04 | 1.60E-04 | 2.25E-04 | 0.00E+00 | 0.00E+00 | 3.17E-02 | 4.43E-02 | 1.16E-05 | 1.62E-05 | 4.59E-04 | 6.42E-04 | 4.61E-04 | 6.45E-04 | 7.56E-06 | 1.06E-05 |
|  | 14 | 8.22E-05 | 1.15E-04 | 1.42E-04 | 1.99E-04 | 1.19E-04 | 1.66E-04 | 2.61E-02 | 3.65E-02 | 1.12E-05 | 1.56E-05 | 3.83E-04 | 5.36E-04 | 4.37E-04 | 6.12E-04 | 7.39E-06 | 1.03E-05 |
|  | 15 | 7.99E-05 | 1.12E-04 | 1.51E-04 | 2.11E-04 | 0.00E+00 | 0.00E+00 | 2.78E-02 | 3.90E-02 | 1.29E-05 | 1.81E-05 | 4.39E-04 | 6.15E-04 | 4.12E-04 | 5.77E-04 | 7.44E-06 | 1.04E-05 |
|  | 16 | 6.06E-05 | 8.48E-05 | 1.71E-04 | 2.40E-04 | 1.38E-04 | 1.93E-04 | 2.23E-02 | 3.12E-02 | 1.51E-05 | 2.11E-05 | 3.64E-04 | 5.10E-04 | 4.71E-04 | 6.59E-04 | 7.57E-06 | 1.06E-05 |
|  | 17 | 6.38E-05 | 8.93E-05 | 1.62E-04 | 2.27E-04 | 0.00E+00 | 0.00E+00 | 2.93E-02 | 4.11E-02 | 1.32E-05 | 1.85E-05 | 4.44E-04 | 6.21E-04 | 4.82E-04 | 6.75E-04 | 7.48E-06 | 1.05E-05 |
|  | 18 | 9.44E-05 | 1.32E-04 | 1.50E-04 | 2.10E-04 | 0.00E+00 | 0.00E+00 | 2.69E-02 | 3.77E-02 | 1.19E-05 | 1.67E-05 | 4.05E-04 | 5.67E-04 | 5.89E-04 | 8.25E-04 | 7.36E-06 | 1.03E-05 |
|  | 19 | 5.12E-05 | 7.16E-05 | 1.56E-04 | 2.19E-04 | 1.27E-04 | 1.78E-04 | 2.97E-02 | 4.15E-02 | 1.22E-05 | 1.70E-05 | 4.40E-04 | 6.17E-04 | 4.82E-04 | 6.75E-04 | 7.50E-06 | 1.05E-05 |
|  | 20 | 4.61E-05 | 6.45E-05 | 1.59E-04 | 2.22E-04 | 0.00E+00 | 0.00E+00 | 2.84E-02 | 3.97E-02 | 1.34E-05 | 1.88E-05 | 3.82E-04 | 5.35E-04 | 4.96E-04 | 6.94E-04 | 7.02E-06 | 9.83E-06 |
| **Min.** | | 4.61E-05 | 6.45E-05 | **9.60E-05** | **1.34E-04** | **0.00E+00** | **0.00E+00** | **1.92E-02** | **2.69E-02** | **9.87E-06** | **1.38E-05** | **3.53E-04** | **4.94E-04** | **3.97E-04** | **5.56E-04** | **6.15E-06** | **8.61E-06** |
| **Max.** | | 1.20E-04 | 1.68E-04 | **1.71E-04** | **2.40E-04** | **1.46E-04** | **2.04E-04** | **3.17E-02** | **4.43E-02** | **2.22E-05** | **3.11E-05** | **4.72E-04** | **6.61E-04** | **5.89E-04** | **8.25E-04** | **9.60E-06** | **1.34E-05** |
| **Mean** | | 8.57E-05 | 1.20E-04 | **1.42E-04** | **1.99E-04** | **7.05E-05** | **9.87E-05** | **2.63E-02** | **3.69E-02** | **1.45E-05** | **2.03E-05** | **4.15E-04** | **5.81E-04** | **4.60E-04** | **6.44E-04** | **7.24E-06** | **1.01E-05** |

**Table 7 (Suppl.):** **Calculated carcinogenic chronic daily intake (CDI_Dermal_) and incremental life time cancer risk (ILCR_Dermal_) values for male and female due to dermal exposure of Pb, Ni and Cd to the soils of brinjal-producing areas of Jamalpur district, Bangladesh**

| **Name of Upazila** | **Sample ID** | **Carcinogenic chronic daily intake (CDI_Dermal_)** | | | | | | **Incremental life time cancer risk (ILCR_Dermal_)** | | | | | |
| --- | --- | --- | --- | --- | --- | --- | --- | --- | --- | --- | --- | --- | --- |
|  |  | **Pb** | | **Ni** | | **Cd** | | **Pb** | | **Ni** | | **Cd** | |
|  |  | **Male** | **Female** | **Male** | **Female** | **Male** | **Female** | **Male** | **Female** | **Male** | **Female** | **Male** | **Female** |
| Melandaha | 1 | 3.86E-08 | 5.40E-08 | 5.24E-08 | 7.34E-08 | 1.32E-09 | 1.85E-09 | 1.09E-09 | 1.53E-09 | 1.19E-06 | 1.67E-06 | 7.93E-07 | 1.11E-06 |
|  | 2 | 4.03E-08 | 5.65E-08 | 5.75E-08 | 8.05E-08 | 1.56E-09 | 2.18E-09 | 1.14E-09 | 1.60E-09 | 1.31E-06 | 1.83E-06 | 9.33E-07 | 1.31E-06 |
|  | 3 | 2.38E-08 | 3.34E-08 | 5.01E-08 | 7.02E-08 | 1.28E-09 | 1.79E-09 | 6.75E-10 | 9.45E-10 | 1.14E-06 | 1.60E-06 | 7.69E-07 | 1.08E-06 |
|  | 4 | 3.77E-08 | 5.28E-08 | 4.31E-08 | 6.04E-08 | 1.52E-09 | 2.13E-09 | 1.07E-09 | 1.49E-09 | 9.81E-07 | 1.37E-06 | 9.12E-07 | 1.28E-06 |
|  | 5 | 4.20E-08 | 5.88E-08 | 4.83E-08 | 6.76E-08 | 1.19E-09 | 1.66E-09 | 1.19E-09 | 1.67E-09 | 1.10E-06 | 1.54E-06 | 7.11E-07 | 9.96E-07 |
|  | 6 | 3.46E-08 | 4.85E-08 | 5.44E-08 | 7.61E-08 | 1.30E-09 | 1.82E-09 | 9.79E-10 | 1.37E-09 | 1.24E-06 | 1.73E-06 | 7.82E-07 | 1.09E-06 |
|  | 7 | 5.27E-08 | 7.37E-08 | 3.91E-08 | 5.48E-08 | 0.00E+00 | 0.00E+00 | 1.49E-09 | 2.09E-09 | 8.90E-07 | 1.25E-06 | 0.00E+00 | 0.00E+00 |
|  | 8 | 4.95E-08 | 6.93E-08 | 3.29E-08 | 4.61E-08 | 0.00E+00 | 0.00E+00 | 1.40E-09 | 1.96E-09 | 7.49E-07 | 1.05E-06 | 0.00E+00 | 0.00E+00 |
|  | 9 | 5.54E-08 | 7.76E-08 | 4.04E-08 | 5.66E-08 | 0.00E+00 | 0.00E+00 | 1.57E-09 | 2.20E-09 | 9.19E-07 | 1.29E-06 | 0.00E+00 | 0.00E+00 |
| Islampur | 10 | 4.95E-08 | 6.93E-08 | 4.39E-08 | 6.15E-08 | 0.00E+00 | 0.00E+00 | 1.40E-09 | 1.96E-09 | 9.99E-07 | 1.40E-06 | 0.00E+00 | 0.00E+00 |
|  | 11 | 5.43E-08 | 7.60E-08 | 4.35E-08 | 6.10E-08 | 1.56E-09 | 2.18E-09 | 1.54E-09 | 2.15E-09 | 9.91E-07 | 1.39E-06 | 9.36E-07 | 1.31E-06 |
|  | 12 | 4.59E-08 | 6.43E-08 | 4.14E-08 | 5.79E-08 | 1.27E-09 | 1.77E-09 | 1.30E-09 | 1.82E-09 | 9.41E-07 | 1.32E-06 | 7.60E-07 | 1.06E-06 |
|  | 13 | 4.72E-08 | 6.61E-08 | 5.50E-08 | 7.70E-08 | 0.00E+00 | 0.00E+00 | 1.34E-09 | 1.87E-09 | 1.25E-06 | 1.75E-06 | 0.00E+00 | 0.00E+00 |
|  | 14 | 3.80E-08 | 5.33E-08 | 4.89E-08 | 6.84E-08 | 1.27E-09 | 1.78E-09 | 1.08E-09 | 1.51E-09 | 1.11E-06 | 1.56E-06 | 7.64E-07 | 1.07E-06 |
|  | 15 | 3.70E-08 | 5.18E-08 | 5.17E-08 | 7.24E-08 | 0.00E+00 | 0.00E+00 | 1.05E-09 | 1.47E-09 | 1.18E-06 | 1.65E-06 | 0.00E+00 | 0.00E+00 |
|  | 16 | 2.80E-08 | 3.93E-08 | 5.87E-08 | 8.22E-08 | 1.48E-09 | 2.07E-09 | 7.94E-10 | 1.11E-09 | 1.34E-06 | 1.87E-06 | 8.88E-07 | 1.24E-06 |
|  | 17 | 2.95E-08 | 4.13E-08 | 5.55E-08 | 7.77E-08 | 0.00E+00 | 0.00E+00 | 8.35E-10 | 1.17E-09 | 1.26E-06 | 1.77E-06 | 0.00E+00 | 0.00E+00 |
|  | 18 | 4.37E-08 | 6.12E-08 | 5.14E-08 | 7.20E-08 | 0.00E+00 | 0.00E+00 | 1.24E-09 | 1.73E-09 | 1.17E-06 | 1.64E-06 | 0.00E+00 | 0.00E+00 |
|  | 19 | 2.37E-08 | 3.32E-08 | 5.36E-08 | 7.51E-08 | 1.36E-09 | 1.90E-09 | 6.70E-10 | 9.38E-10 | 1.22E-06 | 1.71E-06 | 8.15E-07 | 1.14E-06 |
|  | 20 | 2.13E-08 | 2.98E-08 | 5.45E-08 | 7.62E-08 | 0.00E+00 | 0.00E+00 | 6.03E-10 | 8.44E-10 | 1.24E-06 | 1.73E-06 | 0.00E+00 | 0.00E+00 |
| **Min.** | | **2.13E-08** | **2.98E-08** | **3.29E-08** | **4.61E-08** | **0.00E+00** | **0.00E+00** | **6.03E-10** | **8.44E-10** | **7.49E-07** | **1.05E-06** | **0.00E+00** | **0.00E+00** |
| **Max.** | | **5.54E-08** | **7.76E-08** | **5.87E-08** | **8.22E-08** | **1.56E-09** | **2.18E-09** | **1.57E-09** | **2.20E-09** | **1.34E-06** | **1.87E-06** | **9.36E-07** | **1.31E-06** |
| **Mean** | | **3.96E-08** | **5.55E-08** | **4.88E-08** | **6.84E-08** | **7.55E-10** | **1.06E-09** | **1.12E-09** | **1.57E-09** | **1.11E-06** | **1.56E-06** | **4.53E-07** | **6.34E-07** |

**Table 8 (Suppl.):** **Calculated chronic daily intake (CDI_Oral_) values for male and female due to oral exposure of trace metals from the dietary intake of brinjal fruits collected from different farmers′ field of Jamalpur district**

| **Name of Upazila** | **Sample ID** | **Pb** | | **Ni** | | **Cd** | | **Cr** | | **Cu** | | **Fe** | | **Mn** | | **Zn** | |
| --- | --- | --- | --- | --- | --- | --- | --- | --- | --- | --- | --- | --- | --- | --- | --- | --- | --- |
|  |  | **Male** | **Female** | **Male** | **Female** | **Male** | **Female** | **Male** | **Female** | **Male** | **Female** | **Male** | **Female** | **Male** | **Female** | **Male** | **Female** |
| Melandaha | 1 | 0.021 | 0.030 | 0.0101 | 0.0141 | 0.0055 | 0.0077 | 0.00 | 0.00 | 0.262 | 0.367 | 0.554 | 0.776 | 0.085 | 0.119 | 0.292 | 0.409 |
|  | 2 | 0.030 | 0.042 | 0.0113 | 0.0159 | 0.0043 | 0.0060 | 0.00 | 0.00 | 0.189 | 0.265 | 0.541 | 0.757 | 0.009 | 0.012 | 0.243 | 0.340 |
|  | 3 | 0.035 | 0.049 | 0.0071 | 0.0100 | 0.0046 | 0.0064 | 0.00 | 0.00 | 0.260 | 0.364 | 0.615 | 0.860 | 0.090 | 0.126 | 0.283 | 0.397 |
|  | 4 | 0.040 | 0.057 | 0.0032 | 0.0045 | 0.0049 | 0.0069 | 0.00 | 0.00 | 0.213 | 0.298 | 0.487 | 0.681 | 0.014 | 0.019 | 0.237 | 0.332 |
|  | 5 | 0.029 | 0.041 | 0.0103 | 0.0144 | 0.0000 | 0.0000 | 0.00 | 0.00 | 0.250 | 0.349 | 0.493 | 0.690 | 0.028 | 0.039 | 0.270 | 0.378 |
|  | 6 | 0.032 | 0.045 | 0.0144 | 0.0202 | 0.0000 | 0.0000 | 0.00 | 0.00 | 0.277 | 0.388 | 0.538 | 0.753 | 0.054 | 0.075 | 0.344 | 0.481 |
|  | 7 | 0.076 | 0.106 | 0.0096 | 0.0134 | 0.0000 | 0.0000 | 0.00 | 0.00 | 0.214 | 0.300 | 0.521 | 0.729 | 0.025 | 0.035 | 0.255 | 0.357 |
|  | 8 | 0.056 | 0.078 | 0.0050 | 0.0071 | 0.0042 | 0.0058 | 0.00 | 0.00 | 0.266 | 0.373 | 0.524 | 0.734 | 0.018 | 0.025 | 0.264 | 0.369 |
|  | 9 | 0.070 | 0.098 | 0.0097 | 0.0135 | 0.0000 | 0.0000 | 0.00 | 0.00 | 0.252 | 0.353 | 0.441 | 0.617 | 0.005 | 0.007 | 0.270 | 0.378 |
| Islampur | 10 | 0.066 | 0.092 | 0.0133 | 0.0186 | 0.0000 | 0.0000 | 0.00 | 0.00 | 0.230 | 0.322 | 0.464 | 0.649 | 0.038 | 0.053 | 0.258 | 0.361 |
|  | 11 | 0.050 | 0.069 | 0.0082 | 0.0114 | 0.0034 | 0.0048 | 0.00 | 0.00 | 0.237 | 0.331 | 0.544 | 0.761 | 0.002 | 0.003 | 0.343 | 0.480 |
|  | 12 | 0.046 | 0.064 | 0.0097 | 0.0136 | 0.0000 | 0.0000 | 0.00 | 0.00 | 0.232 | 0.325 | 0.598 | 0.837 | 0.050 | 0.070 | 0.296 | 0.414 |
|  | 13 | 0.069 | 0.096 | 0.0156 | 0.0219 | 0.0000 | 0.0000 | 0.00 | 0.00 | 0.208 | 0.291 | 0.423 | 0.593 | 0.000 | 0.000 | 0.400 | 0.560 |
|  | 14 | 0.041 | 0.058 | 0.0112 | 0.0157 | 0.0000 | 0.0000 | 0.00 | 0.00 | 0.210 | 0.294 | 0.407 | 0.569 | 0.000 | 0.000 | 0.259 | 0.363 |
|  | 15 | 0.035 | 0.050 | 0.0134 | 0.0188 | 0.0000 | 0.0000 | 0.00 | 0.00 | 0.208 | 0.291 | 0.432 | 0.604 | 0.000 | 0.000 | 0.244 | 0.341 |
|  | 16 | 0.026 | 0.036 | 0.0189 | 0.0265 | 0.0045 | 0.0063 | 0.00 | 0.00 | 0.220 | 0.308 | 0.538 | 0.753 | 0.008 | 0.011 | 0.379 | 0.530 |
|  | 17 | 0.051 | 0.071 | 0.0155 | 0.0217 | 0.0000 | 0.0000 | 0.00 | 0.00 | 0.195 | 0.272 | 0.392 | 0.549 | 0.002 | 0.003 | 0.225 | 0.314 |
|  | 18 | 0.053 | 0.074 | 0.0149 | 0.0209 | 0.0000 | 0.0000 | 0.00 | 0.00 | 0.197 | 0.276 | 0.340 | 0.476 | 0.016 | 0.023 | 0.226 | 0.316 |
|  | 19 | 0.031 | 0.043 | 0.0156 | 0.0219 | 0.0064 | 0.0089 | 0.00 | 0.00 | 0.209 | 0.293 | 0.357 | 0.499 | 0.007 | 0.010 | 0.233 | 0.326 |
|  | 20 | 0.040 | 0.056 | 0.0221 | 0.0309 | 0.0000 | 0.0000 | 0.00 | 0.00 | 0.223 | 0.312 | 0.513 | 0.718 | 0.030 | 0.042 | 0.267 | 0.373 |
| **Min.** | | **0.021** | **0.030** | **0.0032** | **0.0045** | **0.0000** | **0.0000** | **0.00** | **0.00** | **0.189** | **0.265** | **0.340** | **0.476** | **0.000** | **0.000** | **0.225** | **0.314** |
| **Max.** | | **0.076** | **0.106** | **0.0221** | **0.0309** | **0.0064** | **0.0089** | **0.00** | **0.00** | **0.277** | **0.388** | **0.615** | **0.860** | **0.090** | **0.126** | **0.400** | **0.560** |
| **Mean** | | **0.045** | **0.063** | **0.0120** | **0.0167** | **0.0019** | **0.0026** | **0.00** | **0.00** | **0.228** | **0.319** | **0.486** | **0.680** | **0.024** | **0.034** | **0.279** | **0.391** |

**Table 9 (Suppl.):** **Calculated hazard quotient (HQ_Oral_) values for male and female due to oral exposure of trace metals from the dietary intake of brinjal fruits collected from different farmers′ field of Jamalpur district**

| **Name of Upazila** | **Sample ID** | **Pb** | | **Ni** | | **Cd** | | **Cr** | | **Cu** | | **Fe** | | **Mn** | | **Zn** | |
| --- | --- | --- | --- | --- | --- | --- | --- | --- | --- | --- | --- | --- | --- | --- | --- | --- | --- |
|  |  | **Male** | **Female** | **Male** | **Female** | **Male** | **Female** | **Male** | **Female** | **Male** | **Female** | **Male** | **Female** | **Male** | **Female** | **Male** | **Female** |
| Melandaha | 1 | 6.06 | 8.48 | 0.51 | 0.71 | 5.49 | 7.69 | 0.00 | 0.00 | 6.55 | 9.16 | 0.79 | 1.11 | 0.61 | 0.85 | 0.97 | 1.36 |
|  | 2 | 8.52 | 11.93 | 0.57 | 0.79 | 4.29 | 6.01 | 0.00 | 0.00 | 4.73 | 6.62 | 0.77 | 1.08 | 0.06 | 0.09 | 0.81 | 1.13 |
|  | 3 | 10.02 | 14.02 | 0.36 | 0.50 | 4.57 | 6.40 | 0.00 | 0.00 | 6.50 | 9.10 | 0.88 | 1.23 | 0.64 | 0.90 | 0.94 | 1.32 |
|  | 4 | 11.53 | 16.15 | 0.16 | 0.23 | 4.93 | 6.90 | 0.00 | 0.00 | 5.32 | 7.45 | 0.70 | 0.97 | 0.10 | 0.14 | 0.79 | 1.11 |
|  | 5 | 8.27 | 11.57 | 0.51 | 0.72 | 0.00 | 0.00 | 0.00 | 0.00 | 6.24 | 8.73 | 0.70 | 0.99 | 0.20 | 0.28 | 0.90 | 1.26 |
|  | 6 | 9.26 | 12.96 | 0.72 | 1.01 | 0.00 | 0.00 | 0.00 | 0.00 | 6.94 | 9.71 | 0.77 | 1.08 | 0.38 | 0.54 | 1.15 | 1.60 |
|  | 7 | 21.67 | 30.34 | 0.48 | 0.67 | 0.00 | 0.00 | 0.00 | 0.00 | 5.35 | 7.49 | 0.74 | 1.04 | 0.18 | 0.25 | 0.85 | 1.19 |
|  | 8 | 15.94 | 22.31 | 0.25 | 0.35 | 4.17 | 5.83 | 0.00 | 0.00 | 6.66 | 9.33 | 0.75 | 1.05 | 0.13 | 0.18 | 0.88 | 1.23 |
|  | 9 | 19.99 | 27.98 | 0.48 | 0.68 | 0.00 | 0.00 | 0.00 | 0.00 | 6.30 | 8.82 | 0.63 | 0.88 | 0.04 | 0.05 | 0.90 | 1.26 |
| Islampur | 10 | 18.81 | 26.34 | 0.66 | 0.93 | 0.00 | 0.00 | 0.00 | 0.00 | 5.76 | 8.06 | 0.66 | 0.93 | 0.27 | 0.38 | 0.86 | 1.20 |
|  | 11 | 14.16 | 19.83 | 0.41 | 0.57 | 3.43 | 4.80 | 0.00 | 0.00 | 5.92 | 8.28 | 0.78 | 1.09 | 0.01 | 0.02 | 1.14 | 1.60 |
|  | 12 | 13.13 | 18.38 | 0.49 | 0.68 | 0.00 | 0.00 | 0.00 | 0.00 | 5.80 | 8.12 | 0.85 | 1.20 | 0.36 | 0.50 | 0.99 | 1.38 |
|  | 13 | 19.60 | 27.45 | 0.78 | 1.09 | 0.00 | 0.00 | 0.00 | 0.00 | 5.20 | 7.29 | 0.60 | 0.85 | 0.00 | 0.00 | 1.33 | 1.87 |
|  | 14 | 11.85 | 16.58 | 0.56 | 0.78 | 0.00 | 0.00 | 0.00 | 0.00 | 5.26 | 7.36 | 0.58 | 0.81 | 0.00 | 0.00 | 0.86 | 1.21 |
|  | 15 | 10.12 | 14.17 | 0.67 | 0.94 | 0.00 | 0.00 | 0.00 | 0.00 | 5.20 | 7.28 | 0.62 | 0.86 | 0.00 | 0.00 | 0.81 | 1.14 |
|  | 16 | 7.32 | 10.25 | 0.95 | 1.32 | 4.47 | 6.25 | 0.00 | 0.00 | 5.50 | 7.71 | 0.77 | 1.08 | 0.06 | 0.08 | 1.26 | 1.77 |
|  | 17 | 14.57 | 20.40 | 0.77 | 1.08 | 0.00 | 0.00 | 0.00 | 0.00 | 4.87 | 6.81 | 0.56 | 0.78 | 0.02 | 0.02 | 0.75 | 1.05 |
|  | 18 | 15.16 | 21.22 | 0.75 | 1.04 | 0.00 | 0.00 | 0.00 | 0.00 | 4.93 | 6.90 | 0.49 | 0.68 | 0.12 | 0.16 | 0.75 | 1.05 |
|  | 19 | 8.78 | 12.29 | 0.78 | 1.09 | 6.36 | 8.90 | 0.00 | 0.00 | 5.23 | 7.32 | 0.51 | 0.71 | 0.05 | 0.07 | 0.78 | 1.09 |
|  | 20 | 11.51 | 16.11 | 1.10 | 1.55 | 0.00 | 0.00 | 0.00 | 0.00 | 5.57 | 7.80 | 0.73 | 1.03 | 0.22 | 0.30 | 0.89 | 1.24 |
| **Min.** | | **6.06** | **8.48** | **0.16** | **0.23** | **0.00** | **0.00** | **0.00** | **0.00** | **4.73** | **6.62** | **0.49** | **0.68** | **0.00** | **0.00** | **0.75** | **1.05** |
| **Max.** | | **21.67** | **30.34** | **1.10** | **1.55** | **6.36** | **8.90** | **0.00** | **0.00** | **6.94** | **9.71** | **0.88** | **1.23** | **0.64** | **0.90** | **1.33** | **1.87** |
| **Mean** | | **12.81** | **17.94** | **0.60** | **0.84** | **1.88** | **2.64** | **0.00** | **0.00** | **5.69** | **7.97** | **0.69** | **0.97** | **0.17** | **0.24** | **0.93** | **1.30** |

**Table 10 (Suppl.): Calculated incremental life time cancer risk (ILCR_Oral_) values for male and female due to oral exposure of Pb, Ni and Cd from the dietary intake of brinjal fruits collected from different farmers′ field of Jamalpur district**

| **Name of Upazila** | **Sample ID** | **Pb** | | **Ni** | | **Cd** | |
| --- | --- | --- | --- | --- | --- | --- | --- |
|  |  | **Male** | **Female** | **Male** | **Female** | **Male** | **Female** |
| Melandaha | 1 | 1.80E-04 | 2.52E-04 | 9.19E-03 | 1.29E-02 | 8.24E-02 | 1.15E-01 |
|  | 2 | 2.53E-04 | 3.55E-04 | 1.03E-02 | 1.44E-02 | 6.43E-02 | 9.01E-02 |
|  | 3 | 2.98E-04 | 4.17E-04 | 6.50E-03 | 9.10E-03 | 6.85E-02 | 9.59E-02 |
|  | 4 | 3.43E-04 | 4.80E-04 | 2.94E-03 | 4.12E-03 | 7.39E-02 | 1.03E-01 |
|  | 5 | 2.46E-04 | 3.44E-04 | 9.35E-03 | 1.31E-02 | 0.00E+00 | 0.00E+00 |
|  | 6 | 2.75E-04 | 3.86E-04 | 1.31E-02 | 1.84E-02 | 0.00E+00 | 0.00E+00 |
|  | 7 | 6.45E-04 | 9.03E-04 | 8.71E-03 | 1.22E-02 | 0.00E+00 | 0.00E+00 |
|  | 8 | 4.74E-04 | 6.64E-04 | 4.59E-03 | 6.43E-03 | 6.25E-02 | 8.75E-02 |
|  | 9 | 5.95E-04 | 8.32E-04 | 8.79E-03 | 1.23E-02 | 0.00E+00 | 0.00E+00 |
| Islampur | 10 | 5.60E-04 | 7.84E-04 | 1.21E-02 | 1.69E-02 | 0.00E+00 | 0.00E+00 |
|  | 11 | 4.21E-04 | 5.90E-04 | 7.42E-03 | 1.04E-02 | 5.15E-02 | 7.21E-02 |
|  | 12 | 3.91E-04 | 5.47E-04 | 8.85E-03 | 1.24E-02 | 0.00E+00 | 0.00E+00 |
|  | 13 | 5.83E-04 | 8.17E-04 | 1.42E-02 | 1.99E-02 | 0.00E+00 | 0.00E+00 |
|  | 14 | 3.52E-04 | 4.93E-04 | 1.02E-02 | 1.43E-02 | 0.00E+00 | 0.00E+00 |
|  | 15 | 3.01E-04 | 4.21E-04 | 1.22E-02 | 1.71E-02 | 0.00E+00 | 0.00E+00 |
|  | 16 | 2.18E-04 | 3.05E-04 | 1.72E-02 | 2.41E-02 | 6.70E-02 | 9.38E-02 |
|  | 17 | 4.34E-04 | 6.07E-04 | 1.41E-02 | 1.97E-02 | 0.00E+00 | 0.00E+00 |
|  | 18 | 4.51E-04 | 6.31E-04 | 1.36E-02 | 1.90E-02 | 0.00E+00 | 0.00E+00 |
|  | 19 | 2.61E-04 | 3.66E-04 | 1.42E-02 | 1.99E-02 | 9.54E-02 | 1.34E-01 |
|  | 20 | 3.42E-04 | 4.79E-04 | 2.01E-02 | 2.81E-02 | 0.00E+00 | 0.00E+00 |
| **Min.** | | 1.80E-04 | 2.52E-04 | 2.94E-03 | 4.12E-03 | 0.00E+00 | 0.00E+00 |
| **Max.** | | 6.45E-04 | 9.03E-04 | 2.01E-02 | 2.81E-02 | 9.54E-02 | 1.34E-01 |
| **Mean** | | 3.81E-04 | 5.34E-04 | 1.09E-02 | 1.52E-02 | 2.83E-02 | 3.96E-02 |

**Table 11: Eigen analysis of the correlation matrix and the PC values of the eigenvectors up to eight variables**

|  | PC1 | PC2 | PC3 | PC4 | PC5 | PC6 | PC7 | PC8 |
| --- | --- | --- | --- | --- | --- | --- | --- | --- |
| Eigenvalue | 4.912 | 3.969 | 2.028 | 1.417 | 1.235 | 1.112 | 0.995 | 0.681 |
| Proportion | 0.273 | 0.221 | 0.113 | 0.079 | 0.069 | 0.062 | 0.055 | 0.038 |
| Cumulative | 0.273 | 0.493 | 0.606 | 0.685 | 0.753 | 0.815 | 0.870 | 0.908 |
| *Variable* |  |  |  |  |  |  |  |  |
| Pb (Brinjal) | -0.312 | -0.248 | -0.219 | 0.069 | -0.099 | -0.068 | -0.166 | 0.070 |
| Ni (Brinjal) | 0.259 | -0.219 | -0.320 | -0.304 | 0.178 | 0.198 | 0.067 | 0.171 |
| Cd (Brinjal) | 0.085 | 0.285 | 0.404 | -0.068 | 0.187 | 0.095 | -0.021 | 0.096 |
| Cu (Brinjal) | -0.121 | 0.396 | -0.240 | 0.073 | -0.150 | 0.072 | -0.027 | 0.011 |
| Mn (Brinjal) | 0.090 | 0.363 | -0.201 | 0.178 | -0.299 | 0.141 | 0.225 | 0.056 |
| Zn (Brinjal) | 0.019 | 0.157 | -0.286 | -0.554 | -0.048 | -0.389 | -0.258 | 0.062 |
| Pb (Soil) | -0.320 | -0.076 | -0.026 | 0.088 | -0.283 | -0.373 | -0.310 | 0.184 |
| Fe (Brinjal) | -0.047 | 0.377 | -0.120 | -0.016 | 0.053 | -0.372 | 0.320 | 0.309 |
| Ni (Soil) | 0.408 | -0.055 | -0.082 | -0.209 | 0.154 | 0.018 | -0.111 | -0.140 |
| Cd (Soil) | 0.152 | 0.287 | 0.368 | -0.237 | -0.066 | -0.312 | 0.140 | -0.147 |
| Cu (Soil) | -0.048 | 0.389 | -0.243 | 0.136 | -0.001 | 0.300 | -0.092 | -0.204 |
| Cr (Soil) | 0.355 | -0.146 | -0.083 | 0.196 | -0.189 | -0.282 | -0.024 | -0.059 |
| Mn (Soil) | 0.052 | -0.226 | -0.114 | -0.157 | -0.533 | 0.148 | 0.514 | 0.102 |
| Zn (Soil) | 0.232 | 0.175 | -0.116 | -0.233 | -0.276 | 0.298 | -0.444 | 0.143 |
| Fe (Soil) | 0.311 | 0.036 | -0.050 | 0.421 | -0.153 | -0.155 | -0.284 | -0.297 |
| OC (Soil) | 0.389 | -0.076 | -0.044 | 0.102 | -0.135 | -0.244 | 0.147 | -0.089 |
| pH | 0.051 | -0.036 | 0.503 | -0.107 | -0.464 | 0.177 | -0.196 | 0.228 |
| EC | 0.276 | 0.007 | -0.015 | 0.335 | 0.212 | -0.005 | -0.090 | 0.745 |
